# Long-term deformation of coastal volcanoes in SE-Asia: linking displacement rates, volcanic activity and flank instabilities

**DOI:** 10.1007/s00445-025-01915-z

**Published:** 2025-12-13

**Authors:** Edgar U. Zorn, Falk Amelung, Francesco Massimetti, Marco Laiolo, Diego Coppola, Thomas R. Walter, Yan Lavallée, Herlan Darmawan

**Affiliations:** 1 Ludwig-Maximilians-Universität München, Theresienstraße 41, 80333 Munich, Germany; 2 GFZ Helmholtz Centre for Geosciences, 14473 Telegrafenberg, Germany; 3 Department of Marine Geosciences, Rosenstiel School of Marine and Atmospheric Sciences, University of Miami, 4600 Rickenbacker Causeway, Miami, FL 33149 USA; 4 Department of Earth Sciences, University of Torino, Via Valperga Caluso 35, 10125 Turin, Italy; 5 Instituto de Geofísica, Universidad Nacional Autónoma de México, C.U., Circuito de La Investigación Científica s/n, Coyoacán, Mexico City, 04150 Mexico; 6 Geophysics Study Program, Department of Physics, Faculty of Mathematics and Natural Sciences, Universitas Gadjah Mada, Yogyakarta, 55281 Indonesia

**Keywords:** Deformation, Flank instability, InSAR, Long-term deformation

## Abstract

Long-term deformation is observed at many volcanoes worldwide, providing valuable insights into sub-volcanic processes. Deformation also informs on volcano flank instability, which presents a major hazard in the event of a complete or partial collapse of the edifice, which may further trigger a tsunami if the volcano is located near the sea. We explore InSAR datasets to investigate surface deformation of 20 potentially hazardous coastal volcanoes in Southeast Asia. We find that over 90% of them exhibit signs of persistent or episodic surface deformation. Most volcanoes experience line-of-sight (LOS) increase with displacement rates spanning a broad range and reaching up to 29 cm/yr at Ruang. These are either steady, or experience distinct acceleration periods, lasting for several months to years following increased volcanic activity measured as the volcanic radiative power (VRP) and reported periods of unrest or eruptions. We attribute the majority of observed deformation to gravity-driven processes and cooling of young surface deposits. Analysis of displacement components shows subsidence for all cases of LOS increase, coupled with varying horizontal displacements showing either (i) convergence, representing inwards displacements of the flanks due to volume loss by gravitational compaction and cooling contraction, (ii) divergence, representing outwards spreading due to instability of the volcano flanks via surficial downslope flank creep or fault sliding or (iii) near-unilateral horizontal displacements across most of the edifice, representing sliding via a deep detachment fault. We suggest that the horizontal component of InSAR deformation on volcanic edifices may be used to quickly assess the dominant deformation patterns. Applying this concept, we identify potential flank instability at four volcanoes (Anak Krakatau, Lewotobi, Sirung and Ulawun), which may pose future collapse hazards. This work offers new insights into the types and rates of volcano flank deformation, demonstrates a direct link between increased volcanic activity levels and deformation rates, and provides an improved comparative basis to other volcanic regions worldwide.

**Supplementary information:**

The online version contains supplementary material available at 10.1007/s00445-025-01915-z.

## Introduction

Flank instabilities at volcanoes pose significant hazards as persistent ground deformation may eventually lead to a partial or full collapse of the edifice. The debris avalanches resulting from collapses can devastate large areas in a single event and cause tsunamis if entering the sea (Paris et al. 2014). Major collapses are a common (near ubiquitous) process during the geological evolution of volcanoes (Siebert et al. 2021; Zernack et al. 2021). Flank instability at volcanoes is generally considered to encompass all scales of downslope deformation with a horizontal component, but also collapse events (Acocella 2021). While collapse events are rapid, long-lasting flank instability may occur due to progressive gravitational flank deformation. Flank collapse (affecting the flanks) and sector collapse (also affecting the conduit and summit of a volcano) are often used synonymously and are known to recur: Volcanoes that have failed have done so repeatedly and are prone to do so again (Belousov et al. 1999; Walter and Schmincke 2002; Cortés et al. 2019; Zernack et al. 2021), which may explain the development of protracted flank deformation. Additionally, episodes of increased volcanic activity expressed as unrest and eruptions may promote accelerations in deformation rates due to shaking by volcanic earthquakes (cf. Lamur et al. 2023) or flank oversteepening by intrusions or deposition of eruptive products (Acocella 2021), potentially jeopardising the structural stability of an edifice and triggering collapse. The December 2018 collapse of the southwest flank of Anak Krakatau (Indonesia) and the associated tsunami that led to > 400 fatalities is a prominent example of a volcano with a history of recurrent collapses (Walter et al. 2019; Williams et al. 2019). Significant flank instability was recorded in the years leading up to the collapse, with increased sliding rates associated with magmatic intrusions and intense eruptive periods (Zorn et al. 2023; Kim et al. 2024). Other examples of flank instability or collapse linked to volcanic activity include the 1980 bulging and subsequent landslide of the northern flank of Mt. St. Helens (USA) due to the intrusion of a cryptodome (Donnadieu et al. 2001). The landslide caused the rapid decompression of magma and fragmentation (e.g., Spieler et al. 2003), triggered a lateral blast that destroyed a 60 km^2^ area and reached up to 23 km away from the volcano (Voight et al. 1983). The volcanic island Stromboli (Tyrrhenian Sea, Italy) experienced a landslide originating from the Sciara del Fuoco sector-collapse scar, involving approx. 30 × 10^6^ m^3^ of material, during an effusive eruption in December 2002, triggering a tsunami (Tinti et al. 2006). The waves with a max runup of over 10 m caused significant damage to buildings along the Stromboli coastline and surrounding islands, reaching various parts of the southern Italian coast (Chiocci et al. 2008). In 2010, an eruption at Pacaya volcano (Guatemala) caused a large portion of the southwestern flank to slump by ~4 m, but slip terminated abruptly without large-scale collapse (Schaefer et al. 2016; Heap et al. 2021). Here, the limited movement was ascribed to rapid deposit consolidation via mechanical clast compaction and repacking (Schaefer et al. 2016), which may have been influenced by changes in overburden and pore pressures due to magmatic intrusions into the edifice. Persistent creep of the flank at Pacaya showed periods of acceleration after eruptions in 2010 and 2014 (Gonzalez-Santana et al. 2022). These recent cases of flank instability evidence the importance of long-term instability and shifts in deformation rate associated with volcanic processes.

Identifying flank instability is often challenging in current datasets as ground motion can be caused by a spectrum of processes, which may not all lead to instability. For example, uplift and subsidence of varying spatial and temporal magnitudes may be related to magmatic intrusion or eruption, respectively (e.g., Bathke et al. 2011; Wauthier and Ho 2024). Cooling and compaction may also cause significant deformation, especially in young eruptive deposits (lavas and volcaniclastic deposits) or above shallow intrusions. Cooling is associated with volumetric contraction of the melt and crystalline phases (Chaussard 2016; Wittmann et al. 2017), volatile resorption (McIntosh et al. 2014) and crystallisation (Caricchi et al. 2014), which promote contractions. Loading and gravitational compaction of new deposits, underlying strata and of lava/magma can cause localised ground subsidence due to pore collapse (Briole et al. 1997; Zorn et al. 2024) and compaction of the vesicular network upon outgassing (Caricchi et al. 2014; Ashwell et al. 2015). Rock alteration and pore pressurisation due to hydrothermal activity may also weaken the rocks that make up the volcanic edifice, easing gravitational spreading of the flanks (Cecchi et al. 2004). Long-term volcano deformation patterns, their underlying causes and their links to volcanic activity are only rarely monitored and explored systematically, despite the inherent threat they pose to the surroundings.

Global remote sensing capabilities have drastically increased over the past decades, with interferometric synthetic-aperture radar data (InSAR) being a particularly suitable tool to detect and quantify volcano deformation and instability (see Poland et al. 2017; Schaefer et al. 2019; Walter 2023 for reviews). Some studies have used InSAR to investigate deformation at a large number of volcanoes (e.g., Becker et al. 2009; Morales Rivera et al. 2016; Albino and Biggs 2021); however, the causes of instability and their interactions with the active volcanic systems remain poorly understood, and insights are limited to select regions. Volcano deformation monitoring using InSAR is particularly challenging in Southeast Asia due to the largely vegetated volcano flanks prevalent in the tropical climate; hence, only limited studies present long-term deformation data in the region. Nevertheless, such data are invaluable to assess future volcano flank collapse and tsunami hazards, considering the high level of activity and young age of the volcanoes present in the area. Therefore, we present new quantitative displacement datasets, using time-series InSAR at 20 volcanoes in Indonesia and Papua New Guinea, to evaluate flank deformation and assess the potential causes, considering common volcanic processes. Our findings offer an improved perspective into the magnitude, timescales and causes of volcano deformation in Southeast Asia and provide an improved basis for identifying instabilities.

## Background on the selected volcanic sites

**Fig. 1:**
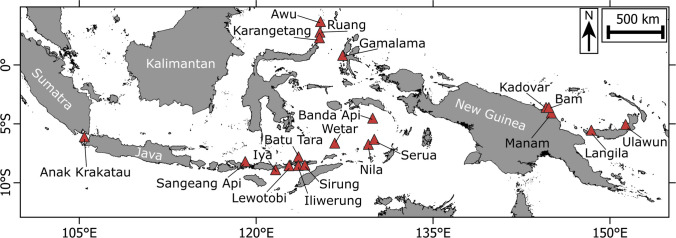
Map of all surveyed volcanoes in Indonesia and Papua New Guinea

The target volcanoes are located in the Sunda, Banda, Halmahera and Sangihe volcanic arcs in Indonesia, and the Bismarck volcanic arc in Papua New Guinea, which are regions with a history of volcanogenic tsunamis (Paris et al. 2014). We prioritised 20 coastal volcanoes that were previously identified to pose a significant tsunami hazard (Fig. 1) based on previous ranking and recommendations (Zorn et al. 2022; Yogaswara et al. 2024). In total, we collected time-series deformation data at 15 volcanoes in Indonesia (Anak Krakatau, Awu, Banda Api, Batu Tara, Gamalama, Iliwerung, Iya, Karangetang, Lewotobi, Nila, Ruang, Sangeang Api, Serua, Sirung and Wetar) and 5 in Papua New Guinea (Bam, Kadovar, Langila, Manam and Ulawun) (Fig. 1).

From Indonesia, Anak Krakatau is the most prominent volcano due to its history of edifice failures and tsunamis. The caldera-forming eruption in 1883 caused a tsunami that resulted in a death toll of nearly 35,000 (Verbeek 1886). A smaller collapse-induced tsunami occurred in 1981 (Paris et al. 2014; Mutaqin et al. 2019) and in 2018 with > 400 fatalities (Walter et al. 2019; Williams et al. 2019). The 2018 collapse was preceded by ground deformation that was interpreted to represent slow flank slip along a deep-seated detachment plane (or décollement), and the collapse was not foreseen as deformation was only identified in hindsight; closer analysis revealed that sliding accelerated upon renewed unrest and eruptive activity (Walter et al. 2019; Zorn et al. 2023; Kim et al. 2024). The detachment fault had accommodated several metres of displacement over more than a decade before it failed catastrophically (Kim et al. 2024). Eruptions and landslides at Awu, Gamalama and Iliwerung have also produced deadly tsunamis in the past (Paris et al. 2014; Mutaqin et al. 2019), and several other volcanoes have been investigated for their potential tsunami impacts including Banda Api (Setyonegoro et al. 2024), Iya (Sakka et al. 2024) or Ruang (Pranantyo et al. 2021). Other volcanoes, such as Batu Tara, exhibit morphologies that indicate clear signs of past collapse events (Laiolo et al. 2018) and a high potential hazard for producing tsunamis (Zorn et al. 2022), but due to the sparsity of data and studies, our records lack clear evidence for the occurrence of tsunamis. This re-emphasises the need for proactive monitoring for both deformation and of coastal volcanoes in this region.

In Papua New Guinea, the most recent tsunami occurred at Kadovar in 2018, possibly caused by the partial collapse of a littoral lava dome during an eruption (Plank et al. 2019). The small volcanic island also displays evidence of a past collapse event revealed by a south-facing scar and submarine debris avalanche deposits (Silver et al. 2009). The most impactful volcano collapse in Papua New Guinea occurred in 1888 at Ritter Island, causing up to 3000 fatalities (Ward and Day 2003; Paris et al. 2014). Ritter Island was not analysed herein as most of the remnant volcano is submerged, with only the rim of the collapse scar rising above sea level.

## Data and methods

### InSAR

We collected Sentinel-1 radar data acquired for 20 volcanoes in Southeast Asia; see Table 1 for a list of volcanoes, observation periods and the radar orbits/tracks used for each. Additionally, the full line-of-sight (LOS) data from all available orbits, as well as the temporal coherence map and interferogram network, are provided in the supplementary material.

**Table 1:** InSAR data overview for all volcanoes in this study. Average LOS deformation rates are calculated for the entire observation period; elevated LOS rates are calculated only for periods of increased deformation, see Fig. 3. A tentative interpretation of the deformation causes is also included

Volcano	Orbit	Observation period	Avg LOS Rate (cm/yr)	Elev. LOS Rate (cm/yr)	Location/notes	Interpretation
Anak Krakatau (post-collapse)	Asc	May 2021–Aug 2024	−4.4	-	Lava flow on the SW-flank	Near-unilateral deformation: Flank instability the lava flow sliding to the SW. The remnant edifice is also deforming, either via detachment fault sliding or surficial deposit compaction
	Desc	May 2021–Aug 2024	−9.2	-	Lava flow on the SW-flank	
Awu	Desc	Jan 2020–Aug 2024	2.1	-	Inside the crater	Deformation inside the crater, but with unclear reference point and therefore not interpreted, see Suppl. Figure S3
Bam	Desc	Jan 2017–Jul 2024	0.1	-	Summit region	No significant deformation
Banda Api	Desc	Mar 2017–Aug 2024	−0.4		N- and S-flank	Very slow deformation; unclear origin
Batu Tara	Asc	Mar 2017–Aug 2024	−0.5	-	Summit region	Cooling and compaction of surface deposits
Gamalama	Desc	Mar 2017–Aug 2024	−2.0	−5.9 Apr 2018–Sep 2019	Summit crater rim	Cooling and compaction or deflation of a shallow hydrothermal reservoir
Iliwerung	Asc	Jan 2017–Mar 2024	−1.0	-	Crater rim	Convergent deformation of the remnant dome: remnant cooling or compaction by incremental rock weakening through hydrothermal alteration
	Desc	Jan 2017–Jul 2024	−1.0	-	Crater rim	
Iya	Desc	Jan 2017–Nov 2024	−1.4	-	Eastern crater rim	Unclear origin
Kadovar	Desc	Jan 2018–Aug 2024	−2.2	−3.3 Jul 2018–Jul 2022	SE-Flank	Cooling and compaction of new deposits and deflation, but flank instability (towards S or SE) may be possible
Karangetang	Desc	Jan 2017–Jun 2024	−7.8	−10.2 Nov 2020–Jun 2024	Summit region	Deflation or cooling and compaction of new deposits, flank instability may be possible
Langila	Asc	Jan 2016–Aug 2024	−5.2	−8.7 Jan 2017–Oct 2020	N-Flank	Convergent deformation: cooling and compaction of the 2017 lavas
	Desc	Nov 2015–Jul 2024	−7.6	−11.1 Jan 2017–Oct 2020	N-Flank	
Lewotobi (Laki-Laki)	Asc	Jan 2017–Nov 2024	−0.7	-	Summit	Near-unilateral edifice deformation: flank instability and likely sliding of the edifice via a detachment fault
	Desc	Jan 2017–Nov 2024	−0.8	-	Summit	
Lewotobi (Perempuam)	Asc	Jan 2017–Nov 2024	1.6	-	Summit	Divergent edifice deformation and uplift: inflation by fluid reservoir recharge
	Desc	Jan 2017–Nov 2024	1.2	-	Summit	
Manam	Desc	Mar 2017–Jun 2024	−3.3	−5.3 Mar 2019–Apr 2022	Upper NE-Flank	The discrepancy in deformation patterns across several flank locations indicates partial flank instability (NE side). Cooling and compaction of new deposits likely add to the deformation
			1.6	-	Lower NE-Flank	
			−2.1	-	SE-Flank	
Nila	Desc	Mar 2017–Jul 2024	−0.5	-	S-Flank	Very slow deformation; possible incremental rock weakening through hydrothermal alteration
Ruang (pre-eruption)	Desc	Jan 2017–Apr 2024	−1.2	−2.5 Dec 2021–Apr 2024	Summit crater rim	Unclear cause, the change in deformation rate is likely related to increasing seismicity
Ruang (post-eruption)	Desc	Apr 2024–Jul 2024	−28.8	-	NE-Flank	Cooling and compaction of deposits from the 2024 eruption
Sangeang Api	Desc	May 2020–Aug 2024	−4.4	-	River valley north of the main cone	Cooling and compaction of deposits in a narrow valley (likely from 2014)
Sirung	Asc	Jan 2017–Nov 2024	−0.6	-	Inner NW caldera wall	Convergent deformation: the opposing caldera walls likely experience flank instability at low sliding rates
	Desc	Jan 2017–Nov 2024	−0.8	-	Inner S caldera wall	
Serua	Desc	Mar 2017–Aug 2024	−0.2	-	Summit region	Very slow deformation; unclear cause
Ulawun	Asc	Mar 2016–Aug 2024	−5.1	−7.4; −6.0 Nov 2019–Jan 2022; May 2024–today	Upper SW-Flank	Divergent edifice deformation: flank instability likely caused by surficial deposit sliding. Cooling and compaction of these deposits likely adds to the deformation
	Desc	Jan 2017–Aug 2024	−8.4	−14.6; −19.2 Nov 2019–Jan 2022; May 2024–today	Upper SW-Flank	
Wetar	Desc	Jan 2017–Aug 2024	1.2	-	Eastern island	Uncertain deformation and reference, therefore not interpreted see Suppl. Figure S27

We created a stack of co-registered Single Look Complex (SLC) data using the Interferometric Synthetic Aperture Radar Scientific Computing Environment (ISCE2) software (Fattahi et al. 2017) and inferred the displacement time series at full synthetic aperture radar (SAR) resolution (~5*20 m^2^) using the phase linking approach, also known as SqueeSAR (Ferretti et al. 2011), as implemented in the open-source Miami Phase Linking software in Python (MiaplPy, Mirzaee et al. 2023). We further used the Miami InSAR Time-series software, developed in Python (MintPy; Yunjun et al. 2019), to enable phase corrections after time-series inversion. We used this to remove the topographic phase with the Copernicus digital elevation model (DEM) in 30 m spatial resolution. We further corrected for phase changes due to tropospheric effects (water vapour variations) by removing predicted phase delays based on the numerical ERA-5 weather model (European Centre for Medium-Range Weather Forecasts, 2019) as implemented in the MintPy workflow. In order to perform the time-series inversion, we selected a network of interferograms for the unwrapping obtained by Delaunay triangulation using temporal and perpendicular baseline thresholds for Delaunay triangles of 120 days and 200 m, respectively, and a ratio for those baselines of 4. Given that in non-coherent areas the temporal coherence can reach 1 by random chance, we apply an additional Gaussian lowpass filter to the temporal coherence to eliminate these pixels. We then use a temporal coherence threshold of 0.75 to select valid pixels (with some exceptions at 0.60 at very active volcanoes). Using the temporal coherence as a filter results in a higher number of reliable persistent points in areas that would otherwise decorrelate due to low spatial coherence caused, for instance, by surface changes (i.e. by emplacement of a lava flow; Yunjun et al. 2019), with the resultant deformation only considered reliable before and after this momentary surface change.

Additional data extraction, processing and plotting were performed using QGIS (v3.38) to generate deformation map plots and Matlab R2024b for extracted point timeseries plots. Individual measurement points for time series evaluation were preferentially picked in the areas with the highest average displacement rates, typically on the flanks and/or the summit of the volcano. We placed the reference points close to, but outside significant detected deformation, though we could not always verify that the reference point reflects stable ground. Our InSAR data can thus detect relative deformation of the volcanic edifices but not larger scale or regional deformation signals (e.g. slow tectonic motion) that cause the reference point to drift over time.

A major challenge in our survey is the scarcity of unvegetated areas that retain sufficient interferometric coherence at C-band. Surface change due to eruptions also reduces coherence. Hence, some volcanoes with frequent/large eruptive activity (e.g. Kadovar, Ruang and Ulawun) were additionally processed in separate periods of 2–3 years. In each period, the same approximate location is selected for time-series analysis, and the LOS displacements of subsequent periods are considered sequentially.

For volcanoes where we could obtain time-series data in both ascending (asc) and descending (desc) orbit geometries for the same time period, we additionally performed a decomposition to resolve vertical (dV; up/down) and horizontal (dH; east/west) ground displacements in MintPy after Wright et al. (2004). We extracted these ground displacements during discrete time intervals overlapping between the two viewing geometries.

### Volcanic activity level: integrating eruption reports and volcanic radiative power

We supplement our quantitative deformation analysis with an assessment of activity monitored at each volcano. This is done in two folds. Where available, we use the thermal anomalies detected by the MIROVA system (Middle InfraRed Observation of Volcanic Activity; Coppola et al. 2016; 2023) as the volcanic radiative power (VRP) has proven to be a reliable indicator of ongoing surface activity. It approximately scales with the presence of new lava at the surface, increased explosivity and/or high-temperature gas emissions (see Coppola et al. 2023 and references therein). This analysis relies on the MIR excess radiance from a thermal anomaly acquired worldwide with a temporal resolution of two images per night using the MODIS sensor to estimate the VRP from selected hotspot pixels on the volcano. It is important to note that VRP datasets may be affected by clouds, volcanic plumes, and unfavourable geometry of the satellite image acquisition angle, possibly masking or overlooking thermal anomalies and underestimating VRP values (Coppola et al. 2023).

Due to limitations in data processing, not all volcanoes are routinely monitored by MIROVA. Here, VRP data are available for 10 volcanoes (Anak Krakatau, Batu Tara, Kadovar, Karangetang, Langila, Lewotobi, Manam, Ruang, Sangeang Api, and Ulawun). To fill gaps in VRP records and assess the activity of volcanoes not covered by MIROVA, we further considered activity data via reports of unrest and eruptive activity, including mentions of the explosive volcanic index (VEI) of eruptions, using the database of the Global Volcanism Program (GVP; Global Volcanism Program 2024a), although these can only be used qualitatively. Here, we refer to increased volcanic activity levels as periods of elevated VRP (reflected by peaks associated with surface heat flux emission) and to instances of reported unrest or eruptions.

## Results

**Fig. 2:**
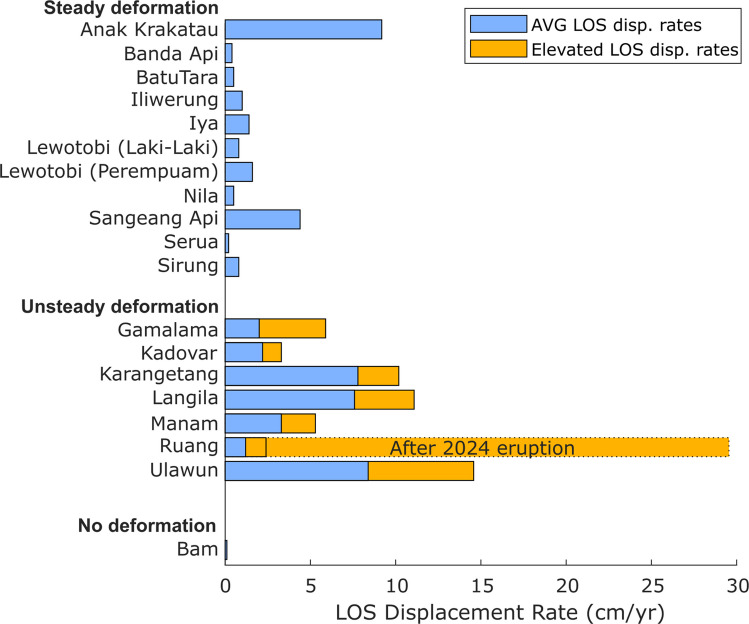
Averaged LOS displacement rates for the analysed volcanoes (excl. Awu and Wetar). Orange bars additionally show the elevation of LOS displacement rates due to volcanic activity in the same locations. For Ruang, the post-eruption bar is shown as a dashed outline as it had to be picked in a different location than the pre-eruption deformation

We find surface deformation at 18 out of 20 volcanoes, mostly expressed as steady or episodic displacements in LOS (Table 1). For each processed dataset, detailed information (track number, coherence map, interferogram network) is provided in the supplementary material (Figs. S1–S27). Note the convention that LOS increase or decrease refers to the radar range, meaning the distance from sensor to ground. Hence, a LOS increase is plotted as negative displacements as the ground moves away from the satellite sensor. Averaged displacement rates for all volcanoes are approximated using linear intervals and vary strongly between near zero and several tens of centimetres per year (Fig. 2). The highest displacement rates are found at Ruang directly following the April 2024 eruption, showing LOS increase with an average of ~29 cm/yr until January 2025. LOS decreases (i.e. displacements towards the satellite sensor) are only observed in a few instances but none exceeding ~2 cm/yr.

**Fig. 3:**
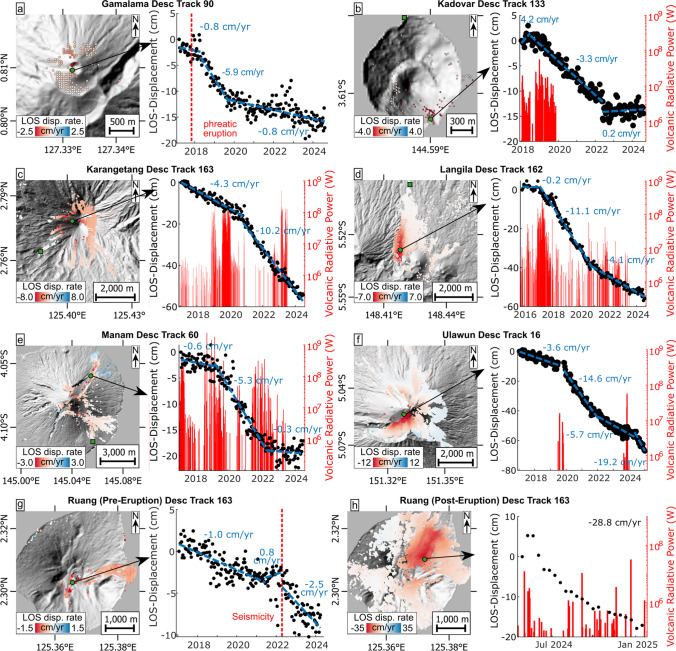
Map plots and corresponding time series displacements of select points on volcanoes with unsteady LOS deformation rates alongside volcanic activity: **a** Gamalama, **b** Kadovar (the map showing the June 2018 to June 2020 period, see Suppl. Figure S11 for the other periods), **c** Karangetang, **d** Langila, **e** Manam, **f** Ulawun, **g** Ruang before the April 2024 eruption and **h** Ruang after the April 2024 eruption. Volcanic activity is plotted in red (as VRP) or is annotated (reported observations from the GVP catalogue) in red dashed lines. Displacement rates are shown for select linear intervals in blue dashed lines. Sampling points are marked as green points and reference points as green squares (refer to Suppl. Figures for all reference point locations)

**Fig. 4:**
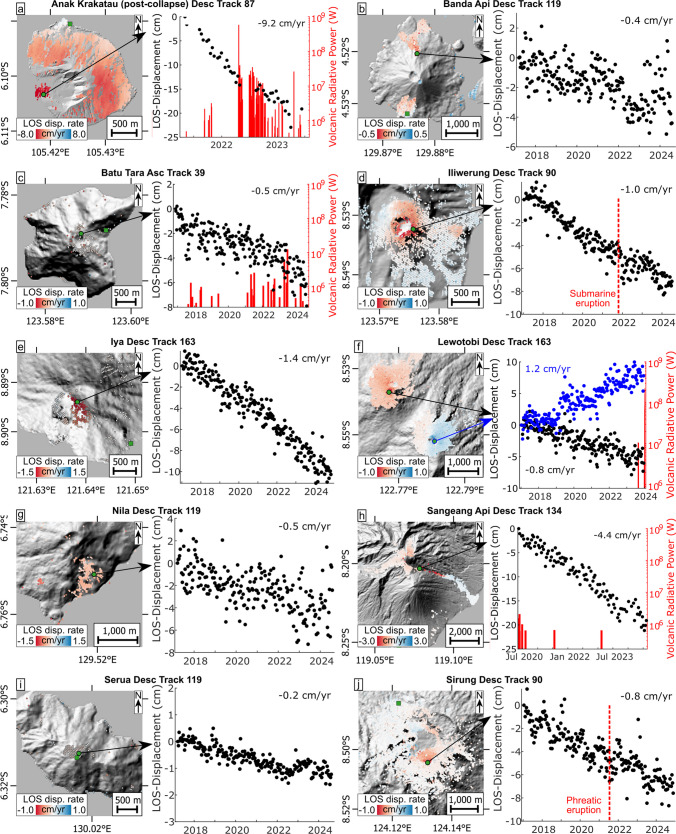
Map plots and corresponding time series displacements of select points on volcanoes with steady (i.e. near linear) LOS deformation rates: **a** Anak Krakatau, **b** Banda Api, **c** Batu Tara, **d **Iliwerung, **e** Iya, **f** Lewotobi, **g** Nila, **h** Sangeang Api, **i** Serua and **j** Sirung. Volcanic activity is plotted in red (as VRP) or is annotated (reported observations from the GVP catalogue) in red dashed lines. Averaged displacement rates are shown as an average for the entire observation period in black. Sampling points are marked as green points and reference points as green squares (refer to Suppl. Figures for all reference point locations)

We broadly classify the observed deformation into two distinct regimes:

1. *Unsteady deformation* refers to volcanoes that experience sudden changes in displacement rates associated with increased volcanic activity levels (Fig. 3).
2. *Steady deformation* refers to volcanoes that maintain steady and linear LOS displacement rates throughout the observation period, irrespective of volcanic activity (Fig. 4).

Volcanoes exhibiting unsteady deformation include Gamalama, Kadovar, Karangetang, Langila, Manam, Ulawun and Ruang. They all exhibit sudden accelerations in LOS displacement rates. Prior to the changes, steady LOS increase (at rates < 5 cm/yr) is almost always observed, except for Kadovar, which first experienced LOS decrease at 4.2 cm/yr. Following periods of increased volcanic activity levels, we observed accelerations in LOS increase in all cases (Fig. 3). These elevated displacement rates vary strongly in magnitude (2.5 to 14.6 cm/yr) and last multiple years; the shortest period of elevated LOS increase being ~1.5 years at Gamalama (Fig. 3a). At Langila, Ulawun and Ruang, these elevated displacement rates appear to decay over time (Fig. 3d, f and h), while constant (elevated) displacement rates are observed at Gamalama, Kadovar, Karangetang, Manam and Ruang (Fig. 3a–c, e and g). At Gamalama, Kadovar and Manam, the elevated displacement rates eventually appear to reduce abruptly before the end of the observation period (Fig. 3a, b and e).

Volcanoes with steady deformation include Anak Krakatau, Banda Api, Batu Tara, Iliwerung, Iya, Lewotobi, Nila, Sangeang Api, Serua and Sirung (Fig. 4). These are all characterised by LOS increase at constant displacement rates, ranging between 0.2 and 9.2 cm/yr. Only Lewotobi (Perempuan edifice) shows LOS decrease, constrained at 1.2 cm/yr. From these volcanoes, Anak Krakatau, Batu Tara, Iliwerung, Lewotobi, Sangeang Api and Sirung underwent some volcanic activity within the observation period, but the displacement rates still remained steady. Both Banda Api and Serua showed variabilities in LOS increase rates towards the end of the observation period (from 2023 onwards; Fig. 4b and i); however, these fluctuations do not appear to change the overall deformation trend significantly. At Banda Api, we advance that this variation may reflect the high levels of noise in the dataset.

No significant deformation was detected at Bam volcano and only very slow deformation (< 0.2 cm/yr) was observed at Serua. However, in both cases, the small size of high-coherence areas limited the choice of reference point, thus potentially masking some deformation. Hence, we cannot reliably distinguish whether there is no deformation occurring or whether it cannot be detected due to the small area of coherence. Surface deformation was also analysed at Awu and Wetar volcanoes, but the data quality was deemed insufficient to provide reliable ground deformation constraints due to poor coherence and lack of reliable referencing points. The crater at Awu volcano likely underwent some deformation (deformation coincided with a remnant lava dome inside the crater), but the magnitude and direction remain unclear. The coherence in the dataset at Wetar was too poor to provide any reliable constraints. Hence, our findings for these volcanoes are only presented in the supplementary material (Figs. S3, S27) and we do not discuss them further.

## Data description and interpretation for individual volcanoes

The following section provides a general description of volcanic activity levels, coherent data coverage and deformation for each volcano. We further interpret the deformation patterns and speculate on the driving mechanisms.

### Unsteady deformation volcanoes

#### Gamalama, Indonesia

The volcanic activity over the last decade at Gamalama volcano was characterised by weak explosive activity with one reported VEI 1 eruption in October 2018, which was likely of phreatic origin (Global Volcanism Program, 2018a). For our InSAR timeseries between March 2017 and August 2024, high temporal coherence is limited to the summit crater as most of the flanks are vegetated. There, deformation initially shows a LOS increase (Fig. 3a) at a rate of 0.8 cm/yr. In October 2018, following the phreatic explosion, displacements accelerated to 5.9 cm/yr before returning to 0.8 cm/yr ~1.5 years later, highlighting the unsteady nature of deformation. The source of the observed ground deformation is unclear; it is unlikely that the phreatic eruption deposited a large volume of deposits, but we cannot rule out potential cooling contraction and compaction of older summit deposits. It is also possible that the LOS increase reflects deflation due to depressurisation of a shallow hydrothermal system after the phreatic eruption, as observed at other volcanoes (e.g., Doke et al. 2021).

#### Kadovar, Papua New Guinea

After centuries of quiescence at Kadovar volcano, an eruptive phase occurred between January 2018 and May 2023 (VEI 2), which was associated with initially high VRP output (up to 60 MW), but no data are available after 2020 (Fig. 3b). The eruption reshaped the southern and eastern flanks of the island, in part due to the emplacement of a littoral lava dome on the eastern shoreline (Plank et al. 2019). Owing to the protracted eruptive period, the InSAR data are processed in four discrete phases to minimise coherence loss during eruptions (January 2018 to June 2018; June 2018 to June 2020; June 2020 to June 2022; and May 2022 to August 2024). The southern flank shows significant unsteady deformation through time, starting with LOS decrease (indicating uplift and/or flank motion) in early to mid-2018 (Fig. 3b). This is followed by LOS increase at 3.3 cm/yr, lasting until mid-2022, when deformation appears to stop (Fig. 3b). From June 2018 onwards, an increase in LOS is also observed on the littoral lava dome, though at lower velocities compared to the southern flank (the dome is not visible in the Hillshade map as the DEM was generated before the eruption; hence, the points appear to be located in the sea; Fig. 3b). The uplift on the south flank during the first months of 2018 can likely be attributed to inflation as magma continued to intrude the edifice during the early phase of the eruption. The following LOS increase likely reflects subsidence deflation and through cooling and compaction of the deposits generated by this eruption, as well as some of the material below. While we cannot rule out wider flank instability as the source of deformation, we believe it is not likely to affect the entire flank as parts of the south-facing remnant collapse scar are not deforming. The LOS increase on the littoral lava may reflect subsidence due to cooling contraction or compaction of weak coastal deposits below the dome.

#### Karangetang, Indonesia

The volcanic activity level at Karangetang volcano was high throughout our observation period (January 2017 to June 2024) and featured frequent eruptions of varying style and intensity (up to VEI 3). Both explosive activity and lava flow emissions occurred, causing VRP values exceeding 100 MW (Fig. 3c). Despite the high activity, coherence remains high across large portions of the entire edifice, showing LOS increase with the highest velocities near the summit and the upper western and southern flanks (Fig. 3c). Displacement rates were unsteady and accelerated in November 2020 following a period of lava production indicated by a peak in VRP, as LOS velocities near the summit increased from 4.3 to 10.2 cm/yr (Fig. 3c). In contrast a later peak in VRP in early 2023 did not coincide with a change in displacement rates. The November 2020 LOS increase in the southwest summit region, associated with the high volcanic activity level and frequent eruptions, points to ground subsidence with an unclear cause. As most of the edifice is affected by this deformation, it is most likely related to deflation, but could also indicate compaction and cooling of widely distributed deposits and/or flank instability.

#### Langila, Papua New Guinea

**Fig. 5:**
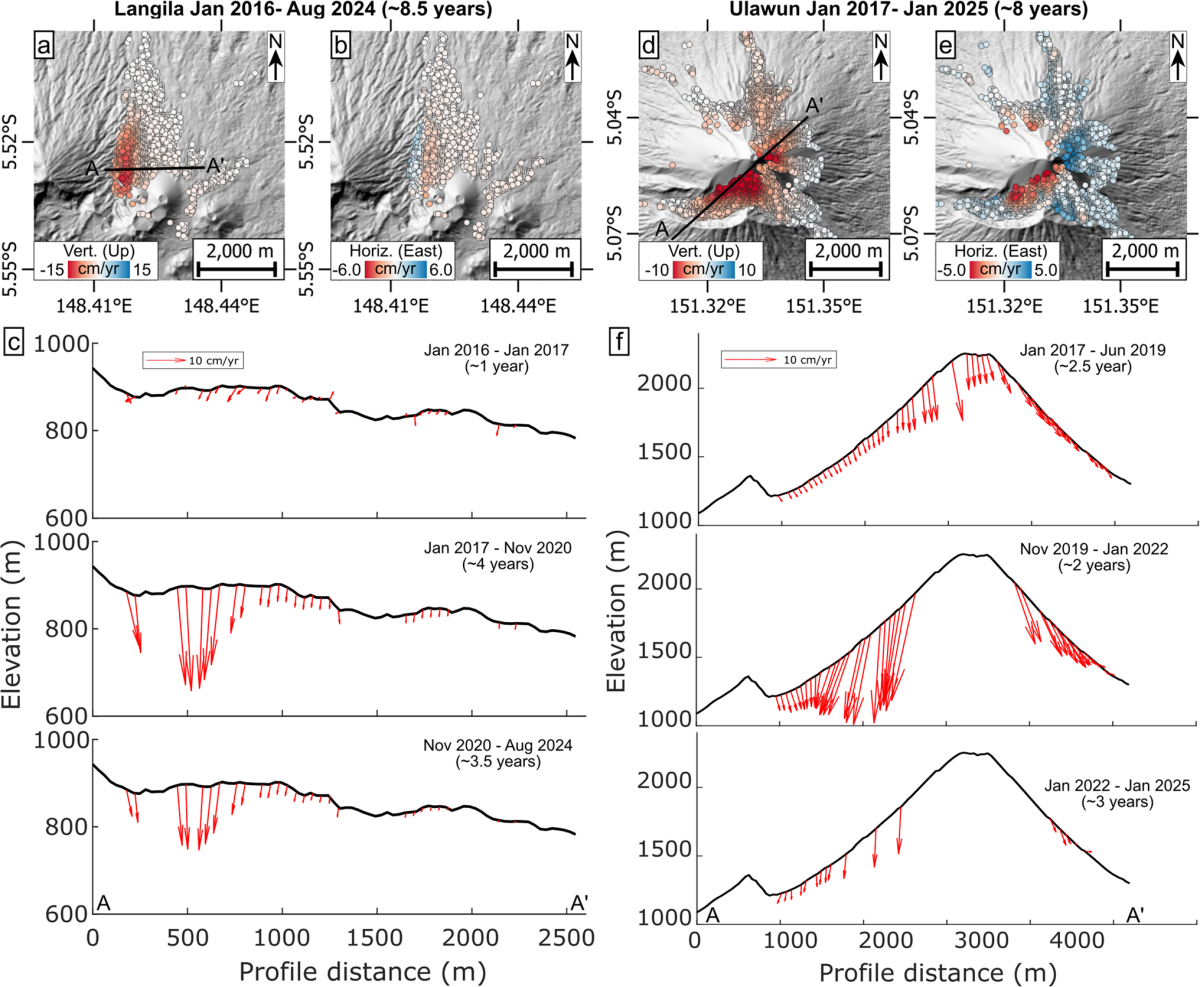
Deformation maps showing the vertical and horizontal components as maps and vectors along elevation profiles for **a**–**c** Langila and **d**–**f** Ulawun. The profiles are further split into time periods before, during and after increases in displacement rates following increased volcanic activity (cf. Fig. 3d, f). Note the SW-NE direction of the profile for Ulawun, so the horizontal E-W component is not fully parallel to the topography

Since April 2016, Langila volcano has been in near-continuous eruption (VEI 2) and the activity level remained high throughout our observation period (November 2015 to August 2024). In early 2017, effusive activity generated a lava flow that descended the northern flank from the summit (Global Volcanism Program, 2020), which led to a particularly high VRP output of up to 300 MW (Fig. 3d). The northern flanks and lava fields that border the edifice of the extinct Talawe volcano in the west exhibit good coherence and are characterised by LOS increase in both ascending and descending orbits, reflecting subsidence (Fig. 3d; Suppl. Figures S13, S14). LOS velocity was unsteady, increasing abruptly to 8.7 cm/yr (asc) and 11.1 cm/yr (desc) in early 2017, and subsequently slowing down progressively to 2.6 cm/yr (asc) and 4.1 cm/yr (desc) over a period of ~7 years. Closer analysis of the vertical and horizontal components of ground displacement signals shows mostly subsidence, with only minor horizontal inward displacement (convergence) of the lava field in E-W direction (Fig. 5a–c). We ascribe this localised convergence and nonlinear reduction in ground subsidence rate to volume loss by progressive cooling contraction of the lava flow and possibly a small component of mechanical compaction of underlying lithologies (e.g. unconsolidated volcaniclastic deposits).

#### Manam, Papua New Guinea

Regular explosive and effusive eruptions of varying intensity were observed throughout the observation period at Manam volcano (March 2017 to June 2024). Of particularly significance is the early stage of a large eruption in August 2018 (VEI 4), which produced pyroclastic density currents and lava flows on the northeast flank (Global Volcanism Program, 2018b). The activity level likely remained elevated throughout with VRPs between 1 and 300 MW, but exceeding 2000 MW in the months after the August 2018 eruption (Fig. 3e), which we assume represents intense and/or near continuous effusion. The InSAR data show coherence on the northeast and southeast flanks. The rest of Manam island is densely vegetated, which prevents ground motion analysis. Both the upper northeast and southeast flanks show LOS increases (Fig. 3e). The upper northeast flank also shows an acceleration in displacement rates to 5.3 cm/yr in late 2018 (Fig. 3e), following the emplacement of pyroclastic deposits and lava flows; however, this coincides with a slight LOS decrease near the coast (Fig. 3e, see also Suppl. Figure S17 for more details), indicating either uplift or horizontal displacements towards the satellite (east). We suggest that the LOS increases likely reflect ground subsidence due to cooling contraction of recent deposits and associated compaction in the subsurface on the upper flanks, whereas the simultaneous LOS increase and decrease in different areas of the northeast flank could indicate flank instability via a listric fault that promotes downslope motion and rotation, although we cannot verify this assertion here. As for the cause of the LOS increase acceleration on the upper northeast flank, the data was insufficient to ascribe whether it is caused by the addition of load from the August 2018 flows or by other factors.

#### Ulawun, Papua New Guinea

Ulawun is a highly active stratovolcano that experienced multiple eruptions during the observation period (March 2016 to August 2024), with a particularly strong (VEI 4) eruption in July to October 2019 (Global Volcanism Program, 2019). This promoted a small VRP peak of ~16 MW. A larger VRP peak of ~60 MW was observed in November 2023, which coincided with a reported VEI 3 eruption. Thus, considering these different eruptive phases, we processed the InSAR data in three discrete periods to minimise coherence loss (January 2017 to June 2019; November 2019 to January 2022; and January 2022 to January 2025). Most of the upper edifice is largely free of vegetation and offers good coherence, with the exception of the western flank. LOS increase occurs near the summit throughout the observation period with the highest velocities just below the crater on the southern side (Fig. 3f, see also Suppl. Figures S25–S26 for more details). The deformation on the upper edifice was unsteady as we observed velocities increase from 2.3 cm/yr (asc) and 3.6 cm/yr (desc) to 7.4 cm/yr (asc) and 14.6 cm/yr (desc), following the 2019 VEI 4 eruption. The higher velocities lasted ~2 years, then slowed progressively. Then, a few months after the November 2023 eruption, the analysis reveals LOS increase, accelerating to 6.0 cm/yr (asc) and 19.2 cm/yr (desc). Minor LOS increase also occurs in a distal deposit north of the edifice. The vertical and horizontal components show primarily subsidence with some minor eastward movement of the entire edifice, even before the 2019 VEI 4 eruption, though deformation rates were highest near the summit on the southwest flank. Following the 2019 eruption, subsidence was additionally accompanied by divergent horizontal displacements across the edifice in the E-W direction (Fig. 5d–f). After ~4 years, displacement rates had reduced, now being primarily vertical on the southwest side with minor horizontal displacements remaining in the northeast (Fig. 5d). The divergent horizontal component of deformation that is observed following the eruption likely indicates downslope deformation due to flank instability. The northeast flank was characterised by downslope deformation throughout the period analysed.

#### Ruang, Indonesia

Ruang volcano explosively erupted in April 2024 (VEI 4), near the end of our observation period (January 2017 to August 2024), which destroyed nearly all the vegetation on the island, leading to the ubiquitous deposition of pyroclastic materials (both pyroclastic density current deposits and tephra) across the island. Thermal analysis only shows any anomaly after the eruption with the appearance of multiple VRP peaks (< 40 MW) over a period of several months, which we ascribe to continued activity (Fig. 3h). Before the eruption, unrest was characterised by weak plume emissions, and seismicity was reported in March to June 2015 (before the observation period) and in April to June 2022 (during the observation period; Global Volcanism Program, 2022a). We processed InSAR data in two periods to image deformation before and after the June 2021 eruption. Before, the island was largely vegetated except near the summit and on a young lava flow on the eastern flank. Near the summit, we observe LOS increase of 1.0 cm/yr until March 2022, when the deformation changed to LOS decrease at 0.8 cm/yr (Fig. 3g). With the onset of seismicity in April 2022, LOS increase resumed at higher velocities of 2.5 cm/yr (Fig. 3g)—a rate which lasted until the start of the eruption. We cannot ascertain the cause of the LOS increase, but the timing indicates a link to the unrest prior to the eventual eruption. Shortly before the seismicity onset, the brief LOS decrease possibly reflects a shallow intrusion of magma into or below the edifice in the build-up of the 2024 eruption.

After the April 2024 eruption, the interferometric coherence was high across the island due to the destruction of the vegetation. We observe an initial LOS increase up to ~29 cm/yr that diminishes over time, with the highest velocities on the upper northeast flank (Fig. 3h), which we ascribed to the likely dual actions of cooling contraction and compaction of the erupted deposits and their substrata. The exceptionally high velocities compared to the deformation signals monitored elsewhere in this study, are likely due to the thickness and the character of the volcaniclastic deposits. They consist largely of tephra and pyroclastic density currents, rather than lava flows, making them highly susceptible to compaction on short-term timescales (Zorn et al. 2024). Some instability of the flank cannot be ruled out, but judging by the extent of the signals observed, it would be restricted to the steep upper slopes on the northwest flank.

### Steady (linear) deformation volcanoes

#### Anak Krakatau, Indonesia

**Fig. 6:**
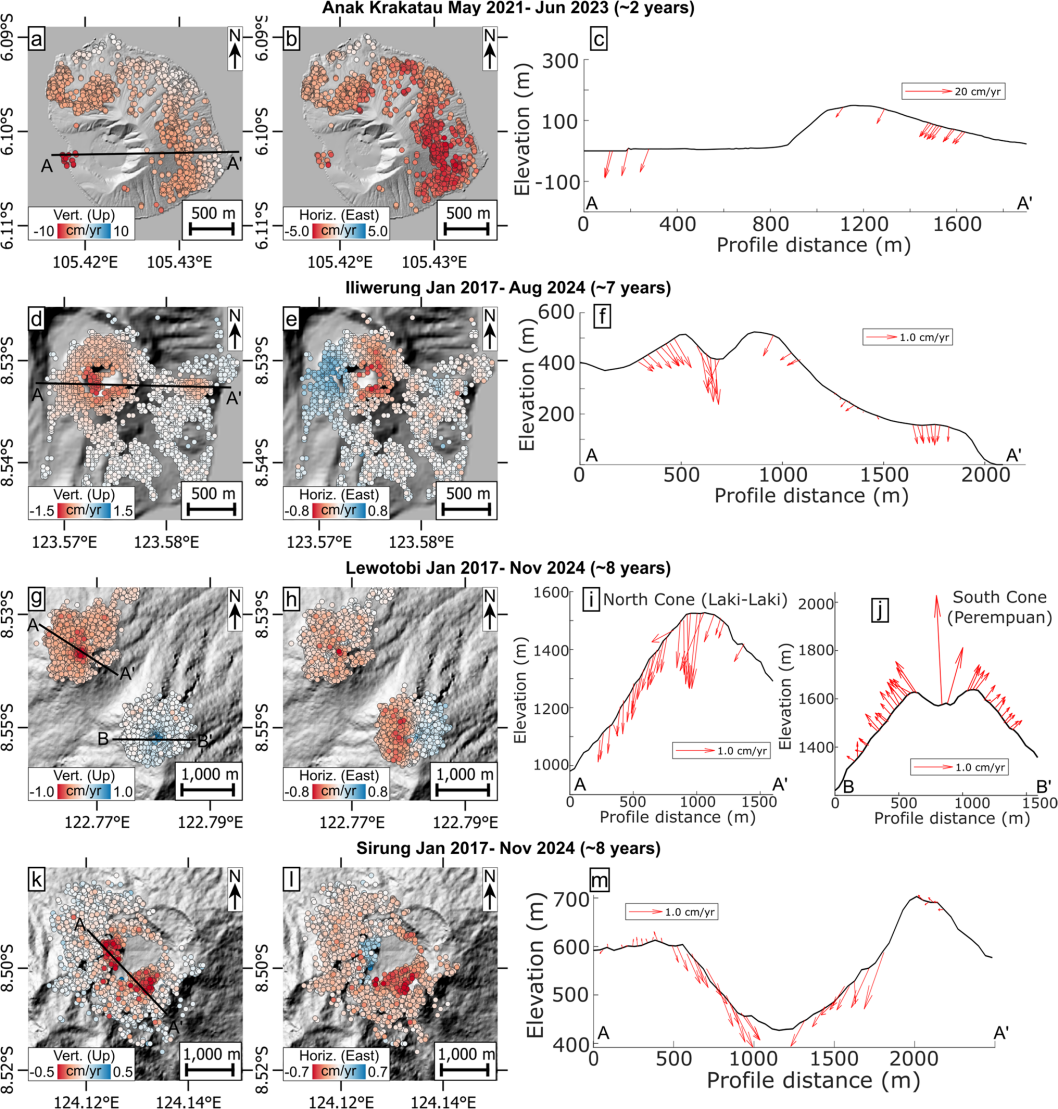
Deformation maps showing the vertical and horizontal components as maps and vectors along elevation profiles for **a**–**c** Anak Krakatau, **d**–**f** Iliwerung, **g**–**j** Lewotobi and **k**–**m** Sirung

The remnant Anak Krakatau island (post 2018 collapse) remained intermittently active with occasional eruptions (VEI 2) throughout the observation period (May 2021 to August 2024). Activity consisted predominantly of minor to moderate explosions and lava effusion, gradually rebuilding the collapsed edifice. This includes the emplacement of a coastal lava flow on the western side of the island in April 2020, which now forms a small lava delta over the 2018 collapse site. The intermittent activity was accompanied by VRP signal fluctuations (Fig. 4a), featuring a distinct, strong peak of ~600 MW and many smaller ones < 40 MW. As the volcano is largely unvegetated, coherence is high across the entire island, except for the active crater area. The volcano shows LOS increases at constant velocities that affect nearly the entire island (Fig. 4a). The highest velocities of 4.4 cm/yr (asc) and 9.2 cm/yr (desc) occur on the coastal lava inside the landslide scar in the southwest (Fig. 4a; see also Suppl. Figures S1, S2). The reference point could only be picked on the island (here near the northern shore); hence, it is not clear whether this location reflects fully stable ground. If deformation also occurs in the north island, the total displacement rates may differ significantly. Vertical and horizontal (EW) displacement components confirm the occurrence of subsidence and also show westwards horizontal deformation of the entire island (Fig. 6a–c). On the remaining eastern flank, these displacements appear to increase from the eastern shore towards the crater (Fig. 6b). The new lava flow emplaced on the western shore has the largest subsidence rates and also shows westwards displacements, but at lower displacements than the remnant edifice (Fig. 6c). The horizontal displacements cannot be explained by cooling and/or compaction alone, implying instability of the newly emplaced lava, or potentially still viscous flow downhill. Potential instability also extends to the remnant edifice as the displacement patterns are similar to those observed prior to the 2018 collapse, although at a much lower rate (LOS of ~9 cm/yr (desc) as opposed to > 50 cm/yr in the months leading to the collapse; Zorn et al. 2023). This may indicate that the new deposits may pose a future collapse hazard for the area, especially if eruptive activity continues to construct a new edifice adding load onto the potentially unstable ground.

#### Banda Api, Indonesia

Banda Api island is largely vegetated, except for lava flows emplaced on the northern and southern flanks in 1988, and for a summit crater, providing sufficient coherence for ground deformation analysis via InSAR (March 2017 to August 2024). LOS velocities are very low (< 0.4 cm/yr or less) on both the flanks, showing a minor LOS increase that appears faster at higher elevations (Fig. 4b, see also Suppl. Figure S5). The timeseries (measured on the upper northern flank) is noisy but overall indicates steady deformation (Fig. 4b). The measured displacement rates are low, but because Sentinel-1 is less sensitive to changes in N-S directions, deformation from these flanks may be missed. The limited possibilities for placing the reference point also present a problem due to the small edifice size. Hence, the point is picked near the southern shoreline in a potentially deforming area, thus masking part of the deformation. Hence, our analysis is unsuitable to address local concerns about flank instability (Setyonegoro et al. 2024).

#### Batu Tara, Indonesia

Batu Tara island was highly active and exhibited frequent explosive activity (incl. strombolian eruptions; VEI 2) until 2016 (Laiolo et al. 2018), with only minor unrest and gas emissions reported since. In our observations (November 2016 to January 2024), this is supported by low VRP values below 3 MW, consistent with the thermal signature of gas emissions; however, a distinct peak reaching ~13 MW occurred in June 2023, but the type of activity was not observed. The volcano features exceptionally steep flanks, particularly on the eastern side, which reach higher angles than the incidence of the radar waves (> 50° slope in parts of the flank). This causes both shadowing and foreshortening on either side of the volcano, restricting deformation measurements to the small summit area only. There, deformation rates are very low (0.5 cm/yr), showing gradual, steady LOS increase (Fig. 4c). Following the June 2023 peak in VRP, we note a slight acceleration in LOS increase, but the noisy displacement data prevent us from identifying any clear changes in the ground deformation rates. The ground displacements likely reflect subsidence due to cooling and/or compaction of erupted deposits emplaced until 2016.

#### Iliwerung, Indonesia

Subaerial eruptions did not take place at Iliwerung volcano during the observation period; however, one submarine eruption occurred in November 2021. Minor vegetation allows broad coherent coverage of the youngest onshore vents. From January 2017 to July 2024, very slow and steady LOS increase at velocities of 1.0 cm/yr (both asc and desc) can be observed on the remains of a lava dome formed in 1870 (Fig. 4d; see also Suppl. Figures S8, S9). These velocities stay constant over the ~7.5 years of measurements, indicating that the submarine eruption did not prompt onshore deformation of the edifice. Ground deformation components reveal the occurrence of subsidence and horizontal convergence of both the inner and outer flanks of the 1870 dome, indicating that this old lava structure is effectively contracting at a slow, steady rate (Fig. 6d–f). Another lava dome erupted in 1948 near the coast also shows signs of subsidence, with no significant horizontal displacements (Fig. 6f). It is possible that the cause for the contraction stems from cooling within the remnant old domes. A landslide also occurred at Iliwerung in 1970, which has been attributed to the degrading effects of hydrothermal alteration at this volcano (Yudhicara et al. 2016), but no further signs of flank instability are found in our data. Hence, we speculate that the slow subsidence observed in both lava domes may also reflect gradual weakening and associated compaction of deposits due to alteration.

#### Iya, Indonesia

Iya volcano has not erupted since 1969, but minor unrest episodes were reported in 2016 (just before the observation period; January 2017 to November 2024) and in January 2024, and stronger unrest was observed in October 2024 (at the end of the observation period), mainly consisting of steam emissions and seismic activity. The vegetation is sparse around the crater, showing some interferometric coherence that indicates a slow, constant LOS increase at 1.4 cm/yr, primarily on the eastern side (Fig. 4e). The origin of these displacements is unclear; however, we note several similarities to Iliwerung in terms of the small size of the edifice, the close proximity to the sea and the similar displacement rates. This suggests a similar underlying cause, here interpreted as remnant cooling and gradual weakening of rocks due to hydrothermal alteration, but this cannot be further substantiated here.

### Lewotobi, Indonesia

Lewotobi volcano consists of two adjacent edifices: Laki-Laki in the northwest of our imaging area and Perempuam in the southeast. Lewotobi last erupted in 2003 (before our survey; January 2017 to November 2024) with a possible (but uncertain) eruption listed in 2014 in the GVP. Increased seismicity was recorded in December 2023, followed rapidly by eruptive activity at Laki-Laki (VEI 2). The 2024 eruptive activity featured intermittent, yet persistent lava emissions and explosive activity, which culminated in a major explosive event on November 3rd (at the end of our observation period), which generated pyroclastic density currents and caused fatalities (Global Volcanism Program 2024b). This is reflected in our data with a VRP output exceeding 200 MW. The upper edifices of Lewotobi both exhibit high coherence and show contrasting deformation patterns through the observation period, with Laki-Laki featuring LOS increase at low but steady velocities of 0.7 cm/yr (asc) and 0.8 cm/yr (desc), and Perempuam showing steady LOS decrease at slightly higher velocities of 1.6 cm/yr (asc) and 1.2 cm/yr (desc), all measured near the respective summit (Fig. 4f). Ground deformation components reveal inflation of Perempuam due to uplift and outward spreading of the upper edifice, strongly indicating inflation likely caused by magma intrusion or fluid pressurisation (Fig. 6g, h and j). In contrast, Laki-Laki was subsiding in the years prior to the 2024 activity; the horizontal component indicates slow westwards movement of the entire edifice (Fig. 6g–i). This instability may be explained by slip along a detachment fault underneath the edifice, which may qualify Laki-Laki as a potential future collapse hazard, particularly in light of the continuing eruptive activity at this volcanic centre.

#### Nila, Indonesia

No activity data or reports exist for Nila Island since its last eruption in 1968. Coherent coverage is very limited due to the dense vegetation, but some small unvegetated patches can be found on the south and southeast flanks that permit deformation tracking. Between March 2017 and July 2024, these exhibit slow and steady LOS increase (Fig. 4g), but the velocities are very low (~0.5 cm/yr). Here, the selected reference point is also a potential source of error as it is picked within a small isolated point cluster in the north of the island, which may not be very reliable but lacks alternative locations. Bright ground discolouration of unvegetated flank portions is visible on satellite images on the Southeast flank (Zorn et al. 2022), indicating the presence of hydrothermal alteration, which may have weakened rocks that make up the flank; thus, the deformation pattern may hint at potential instability, but our analysis cannot provide any further information.

#### Sangeang Api, Indonesia

Eruptions during the observation period at Sangeang Api volcano (May 2020 to August 2024) consist of weak to moderate explosive activity (Global Volcanism Program, 2022b), but a large explosion in 2014 (VEI 4) generated pyroclastic density current deposits and tephra around the active central cone. The VRP remained low, with occasional peaks below 2 MW (Fig. 4h). The southern cone exhibits poor interferometric coherence (despite seemingly sparse vegetation), but the active central cone and the surrounding eruptive deposits are partially coherent. These areas mostly show very minor LOS increase, but inside a narrow valley extending eastwards from the cone, steady LOS increase reached up to 4.4 cm/yr during the ~ 4-year observation period (Fig. 4h). The poor coherence of the displacements limits interpretation, but it is likely that cooling and/or compaction of the 2014 deposits carried on during the entire survey as these reach thicknesses of several metres in confined valleys.

#### Serua, Indonesia

No activity reports exist for the small volcanic island of Serua, which underwent its last known eruption in 1921. Most of the island is vegetated, with only the summit crater having sufficient coherence for deformation measurements. Between March 2017 and August 2024, deformation is detected with a minor LOS increase at a steady rate ~0.2 cm/yr (Fig. 4i); however, the choice of reference point is in such close proximity to the potential deformation, that it would likely not be stable. Hence, it is possible that some deformation is masked, and we hence do not interpret this deformation further.

#### Sirung, Indonesia

Sirung caldera experienced an eruption in 2015 (before the observation period; January 2017 to November 2024) and a small phreatic eruption in July 2021 (both VEI 1), which was followed by gradually declining seismicity (Global Volcanism Program, 2021). The sparse vegetation enables deformation detection on the inner caldera slopes on the southeast side in descending orbit (Fig. 4j), while data from ascending orbit indicate that the slope on the northwest side is also deforming (see Suppl. Figures S23–S24 for details). On both sides, LOS increase is slow and steady at a rate of ~0.6 cm/yr (asc) and ~0.8 cm/yr (desc). Vertical and horizontal components confirm that both areas move downslope via subsidence and horizontal displacements (Fig. 6k–m). Therefore, both caldera inner walls exhibit flank instability, which may potentially lead to eventual collapse within the caldera. The flanks outside the caldera do not show any deformation.

## Discussion

### Mechanisms of long-term volcano subsidence

**Fig. 7:**
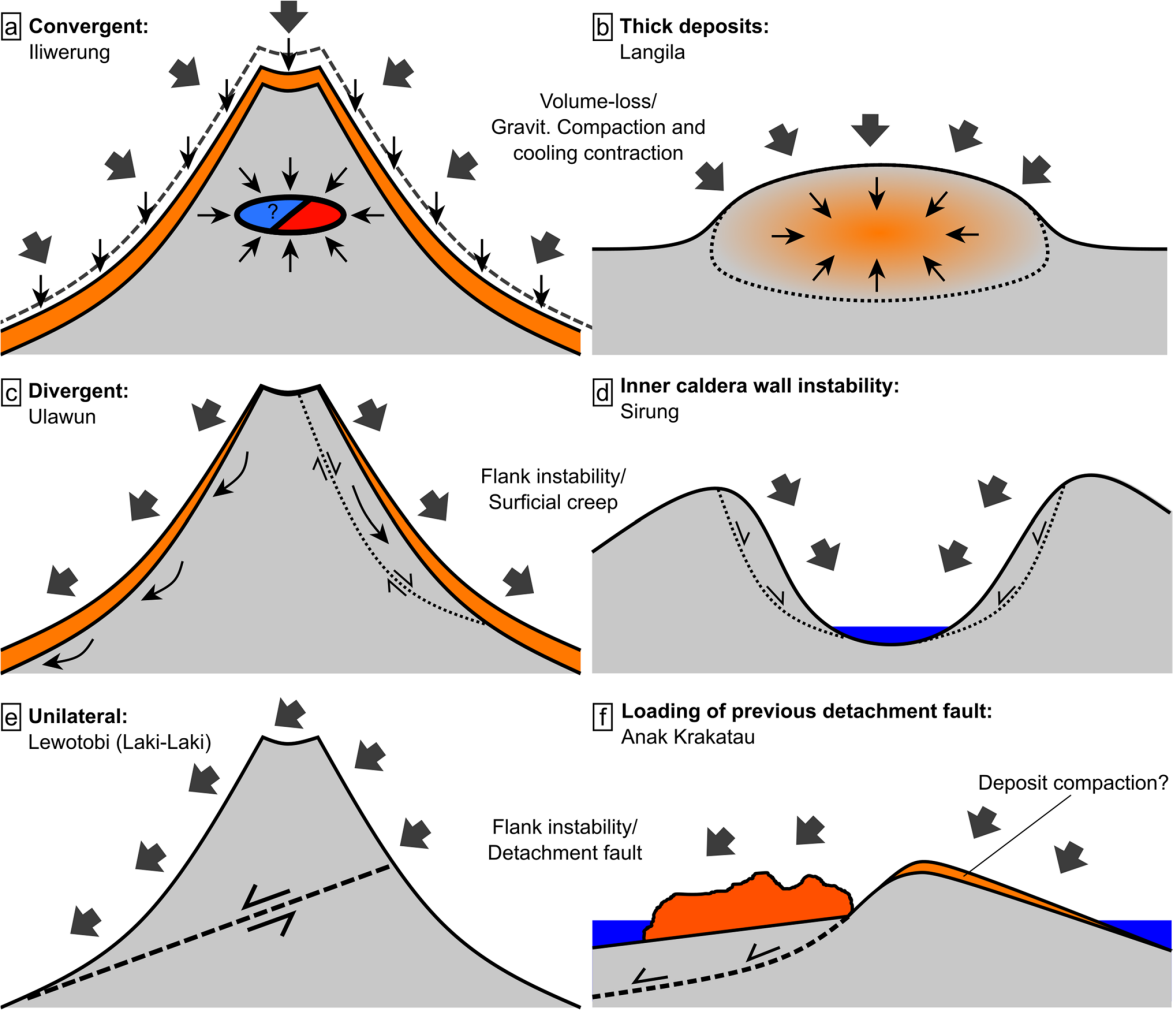
Illustration of the primary long-term volcano deformation mechanisms, causing subsidence. **a** Convergent horizontaldisplacements of a volcanic edifice via volume reduction of cooling and compacting deposits (e.g. Iliwerung) or fluid reservoirs (hydrothermal or magmatic indicated by the blue/red ellipse) and **b** cooling and compaction of a thick volcanic deposit (e.g. a thick lava flow or valley-fill deposits as seen at Langila). **c** Divergent horizontaldisplacements via surficial flank creep, downhill slumping or frictional fault sliding (e.g. Ulawun). **d** The same surficial sliding; however, the horizontal displacements appear convergent as they affect the inner caldera walls (as observed at Sirung). **e** Unilateral horizontal displacements of the entire edifice via a deep-seated detachment fault (décollement; possibly seen at Lewotobi), **f** showing a detachment fault being loaded by eruptive material (as observed at Anak Krakatau)

Many mechanisms may contribute to ground deformation at active volcanoes. In our dataset for SE-Asia, deformation appears to be dominated by gradual LOS increase of the volcanic flanks, edifices and cones of all scales. In most cases, deformation results from vertical ground subsidence, which we can confirm for the volcanoes in all cases with LOS displacements available from both ascending and descending orbits (Fig. 5 and 6). The subsidence always coincides with either (i) inward convergence of the flanks towards the centre of the volcano (at Langila and Iliwerung; Figs. 5a–c and 6d–f), (ii) outward divergence of the flanks from the centre (Ulawun; Fig. 5d–f), or (iii) near-unilateral displacements of the entire edifice (Anak Krakatau and the Laki-Laki edifice of Lewotobi; Figs. 5a–c and 6g–i). We interpret flank convergence and subsidence to indicate volume loss, which may occur by cooling contraction and gravitational compaction of flank deposits or by loss of fluids/fluid pressure in shallow reservoirs (Fig. 7a, b). Divergent spreading displacements and subsidence on the other hand indicate downslope deformation and can therefore be interpreted as volcano flank instability, stemming from either surficial flank creep or slumping from gravitational forces (Fig. 7c). In contrast, unilateral horizontal displacements of large portions of the volcanic edifice can be explained by deep-seated detachment faults (décollement) that accommodate slip (Fig. 7e), thereby indicating larger-scale flank instability.

This ground deformation assessment indicates that horizontal displacements provide highly valuable constraints to help distinguish the possible underlying deformation mechanisms. We advance that the long-term volcano deformation patterns observed in our dataset are likely dominated by gravitational processes (compaction and frictional sliding) and cooling contraction. Frictional sliding can be expected to cause both divergent downslope displacements and unilateral detachment but may pose collapse hazards at varying scales. Therefore, the challenge is to distinguish hazardous flank instability that may result in a collapse of the edifice (or parts of it) from “harmless” instability that may eventually cease without leading to collapse.

### Implications for flank instability and collapse hazards

Using the horizontal component, we identified volcanoes with downslope deformation that may indicate flank instability (Acocella 2021); in our data, these are Anak Krakatau, Lewotobi (Laki-Laki), Sirung and Ulawun.

At Anak Krakatau, the instability is evidenced by the subsidence and westward movement of the new lava flow (Fig. 5a–c), which occurs in the same location as the 2018 collapse, potentially due to reactivation of the same fault upon loading of the new overburden lava. In addition to the new lava, the remnant edifice is also moving westward. Hence, we ponder whether the instability is caused by a deeper décollement below the entire volcanic island. However, as there are some larger westward displacements on the upper slopes of the remnant eastern edifice side (Fig. 5b, c), it could also stem from compaction of recent deposits which would be expected to increase with thickness and therefore edifice height (Fig. 7f). The resulting deformation is therefore caused by either lava flowing or sliding in the west (i.e. divergent displacements; Fig. 7c) combined with compaction of the remnant eastern edifice (i.e. convergent displacements; Fig. 7a) or by bulk sliding of the entire island via a detachment fault (Fig. 7f). While we cannot clearly distinguish between these mechanisms, both imply westward instability at this volcano. As gradual flank motion has been identified as a precursory phenomenon, the observed deformation pattern at Anak Krakatau raises the potential for future collapse and tsunami hazard, especially considering the renewed growth and overburden increase of the edifice during recurrent eruptions.

At Lewotobi volcano, we observed subsidence and near-unilateral westward horizontal displacements of the northern Laki-Laki edifice (Fig. 5g–i). The displacement rates appeared steady prior to the eruption in November 2024, indicating deformation of the entire edifice via a deep-seated detachment fault (Fig. 7e), implying the potential for a future large-volume collapse event. However, the subsidence rate varies, as the summit region shows much larger subsidence rates compared to the lower flanks (Fig. 5i), which may be explained by localised summit deformation from other sources (cooling/compaction/degassing) overprinting the edifice-scale displacements as both flanks otherwise move at approximately the same displacement rate and direction.

At Sirung, the deformation is limited to the inner caldera, which features subsidence and downslope horizontal deformation of two distinct areas on the inner western (moving eastwards) and southeastern (moving westwards) caldera walls (Figs. 5k–m) at steady displacement rates. Hence, these appear as surficial slope instabilities (Fig. 7d). They may be considered future collapse hazards, though as both are directed into the caldera, the resulting landslides are unlikely to be of concern, unless they cause further destabilisation. However, since there is extensive hydrothermal activity and visible alteration within the area, a sudden collapse may cause the sudden unloading of hot groundwater and trigger flash-boiling, resulting in a phreatic explosion (as observed, e.g. in 2012 at Tongariro, New Zealand; Procter et al. 2014).

At Ulawun volcano, we observed subsidence across the entire edifice with horizontal displacements varying through time (Fig. 3d–f). The eastern flank experiences constant downslope displacement, showing a clear onset of divergence after the 2019 VEI 4 eruption (Fig. 3f), likely inducing flank instability. The averaged displacement rates then reduce over time and the deformation becomes primarily vertical (Fig. 3f). Hence, it is likely that the eruption initiated a transient instability on the western side for ~2 years. As the instability on the western flanks initiated with the 2019 eruption, it is likely that the instability is primarily associated with the new surface deposits, but it is also plausible that deeper weak strata have destabilised due to loading from the erupted deposits or were mobilised due to ground shaking. If true, such instabilities may pose a collapse hazard involving minor to moderate amounts of material (new deposits and potentially weak underlying strata), particularly during or following future eruptive activity with high rates of deposition.

In the absence of horizontal displacements, we speculate that the following sectors of volcanoes may perhaps also experience flank instability: the northern flank of Banda Api, the southern flank of Kadovar, the northeast flank at Manam, the southeast flank at Nila and the northeast flank of Ruang after the 2024 eruption. Karangetang also has a very similar deformation pattern in LOS as Ulawun (cf. Fig. 3c, f), where instability is inferred via vertical and horizontal components as downslope deformation. Hence a similar surficial slope instability at Karangetang is possible, but cannot be confirmed here. Most of these are likely surficial downslope movements (Fig. 7c), but the south flank of Kadovar and the northeast flank of Manam exhibited acceleration and deceleration during the observation period. In both cases, cooling and compaction of deposits are likely contributors, but additional mechanisms cannot be ruled out. Nonetheless, the south-facing remnant collapse scar at Kadovar and the evidence for coastal uplift at Manam (possibly indicating listric faulting) still remain concerning. At Ruang (post-April 2024 eruption), most subsidence localised on the upper flanks and did not extend to the coast, likely evidencing compaction and cooling of eruptive deposits rather than flank instability.

**Fig. 8:**
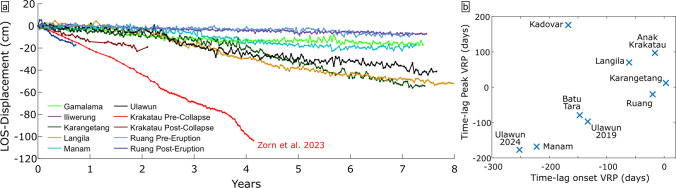
**a** Cumulative LOS displacement over time for select volcanoes (all in descending orbit), showing the large variability of flank deformation rates. For comparison, the LOS flank displacements of Anak Krakatau prior to the 2018 collapse are included using data from Zorn et al. (2023). **b** The time lag between the onset and peak VRP to the changes in deformation rates for individual volcanoes (Anak Krakatau referring to the pre-collapse edifice in 2018)

Finally, further insights on collapse hazards can be drawn from the known critical instability of Anak Krakatau culminating in the major flank collapse on 22nd December 2018. Before its collapse, the island had by far the highest deformation rates compared to all other volcanoes investigated here (Fig. 8a) with an average LOS rate of 17–22 cm/yr, which accelerated to more than 50 cm/yr with increased volcanic activity (Zorn et al. 2023). Next are Ruang post-eruption (~28.8 cm/yr) and Ulawun (~14.6 cm/yr and 19.2 cm/yr, Table 1). Similarly, Karangetang, Langila and Ulawun exhibit particularly high cumulative LOS displacements ranging from 40 to 60 cm within the span of ~7 years (Fig. 8a). However, these magnitudes are still small compared to > 1 m in ~4 years at Anak Krakatau before the Dec 2018 collapse from Sentinel-1 (Zorn et al. 2023) or the ~2 m in ~7 years from CSK (Kim et al. 2024). Hence, it is likely that both the flank displacement rates and the total displacements accumulated over time are important factors for the timing of collapse events, but they likely are not the sole criteria. The lithology of the volcano also plays a major role; for example, laboratory shear experiments have demonstrated that volcanic materials tend to exhibit velocity-weakening behaviour (Kendrick et al. 2014; Laiolo et al. 2018), including Anak Krakatau (Stoepke et al. 2025), which implies that the shear resistance reduces with faster sliding rates, thus enabling rapid rupture progression and potential runaway failure. Weak substrates and pre-existing structures such as past collapse scars and faults have also been suggested to facilitate failure (e.g., Roverato et al. 2021; Marques et al. 2025) and pore-fluid pressure is known to affect failure thresholds (Farquharson et al. 2016). All these factors may influence the surface deformation rates and the geometry of the unstable areas, making it challenging to broadly define volcano flank instability with a quantitative metric.

### Flank acceleration by increased activity

Volcanoes with greater levels of magmatic activity commonly experience higher rates of flank deformation (Poland et al. 2017). Here, we show that 7 of the 20 surveyed volcanoes experienced significant increases in LOS displacement rates following periods of high volcanic activity. This includes Gamalama, Kadovar, Karangetang, Langila, Manam, Ruang and Ulawun. Similar accelerations in conjunction with volcanic activity have been reported at other volcanoes such as Pacaya (Schaefer et al. 2016; Gonzalez-Santana et al. 2022), Etna (Bonaccorso et al. 2011), Stromboli (Di Traglia et al. 2014) or Anak Krakatau (Walter et al. 2019; Zorn et al. 2023; Kim et al. 2024). The exact mechanisms behind this interaction remain poorly understood, but are commonly attributed to stress transfer from intrusions (Walter et al. 2005; Battaglia et al. 2011), surface loading (Odbert et al. 2015), increases in pore fluid pressure (Elsworth and Voight 1996; Hanka et al. 2010) that facilitate fracturing of porous rocks (Farquharson et al. 2016), or oversteepening of flanks by intrusive growth, deposition and possibly shallow inflation (McGuire 1996). Ground shaking by earthquakes may also cause deformation rates to accelerate (Acocella 2021; Lamur et al. 2023). Weakening of flank material through hydrothermal alteration can also affect flank deformation (Heap et al. 2021). Mechanical compaction of unconsolidated volcaniclastic materials is rarely considered as a deformation mechanism at volcanoes. Volcanic activity can promote the deposition of material that increases the overburden and, thus, compaction rates in volcaniclastic materials, but it is expected to rapidly reduce over time. Experimental investigations have shown the significant potential of volcaniclastic lithologies to compact under load, depending on their physical properties and deposit thickness (Zorn et al. 2024). This may partially explain the high deformation rates observed after the eruption at Ruang (Fig. 2h). Such compaction of volcaniclastic materials may also occur in older units and substrata that got buried through time and therefore experience high load stress (Briole et al. 1997), also facilitating downslope creep if deposited on steep topography. The subsidence of the northeast flank at Manam is a likely example of increased displacement rates associated with the deposition of lava on the upper slopes in 2018 (Global Volcanism Program, 2018b), which may explain both increased subsidence through compaction of the underlying strata and/or increased (listric) fault slip of the flank (Fig. 2e).

While the VRP and eruption reports used herein cannot discriminate between these mechanisms, some insight can be drawn from the time lag between the onset of increased volcanic activity (here measured when the VRP increased by at least an order of magnitude above previous background) and the sudden change in deformation rates on the surface. For the volcanoes listed herein, the deformation rates vary between 252 days before and 3 days after the onset VRP (Fig. 8b). This shows that in but one case, the volcano becomes active before the surface deformation rates change, mostly several months before. This is not the same as the peak VRP (measured from the time of the VRP maximum), which has occurred anywhere between 177 days before and 176 days after the change in deformation rates (Fig. 8b). Both onset and peak VRP appear to show a weak linear correlation with one outlier (Fig. 8b; Kadovar). Unfortunately, neither the measured VRP values, nor the displacement rates or their changes show any apparent correlation, though that is unsurprising as both metrics are dependent on additional factors such as the eruption style, lava composition and temperature, edifice geometry or the size of the temperature anomalies. This makes it challenging to compare these values quantitatively, limiting our insights to the time-lag between VRP and deformation rate changes.

Not all volcanic activity in our data resulted in increased displacement rates. Eruptions occurred at both Anak Krakatau and Karangetang that had no observable impact on ground deformation (Figs. 2d and 4a). A similar observation was made by Gonzalez-Santana et al. (2022) at Pacaya, where displacement rates increased during eruptions in 2010 and 2014, but not during similar eruptions in 2007–2009 and 2018–2020. They attribute this increase to additional forcing on the flank through the opening of new eruptive vents, thereby facilitating flank instability. In contrast, an open-vent system can remain stable. It is unclear whether this could apply to either Anak Krakatau or Karangetang as the position of vents appeared stable at both volcanoes. However, new vents formed at Kadovar, where a new littoral lava dome grew over the course of the 2018 eruption (Plank et al. 2019), and Ulawun, where a lava flow erupted on the lower southwest flank shortly after the most intense phase of the 2019 eruption (Global Volcanism Program, 2019). In both instances, significant changes in LOS displacement rates occurred close to the timing of the new vent formations, emphasising the relationship between eruptive activity and deformation.

In our data, elevated LOS displacement rates vary on scales between a few millimetres and several decimetres per year (Table 1). Similar displacement rates have been observed by Poland et al. (2017) for known cases of volcano flank instability, and displacement rates on the order of cm/yr were attributed to cooling and crystallisation of hot surface deposits (lava flows and pyroclastics) (Chaussard 2016; Wittmann et al. 2017), or shallow magma reservoirs (Caricchi et al. 2014). Additionally, compaction of unconsolidated volcaniclastic materials is likely to promote such ground subsidence following eruptions, displaying deformation rates that may initially reach larger magnitudes for short periods of time (Zorn et al. 2024). These contrasting causes make it challenging to recognise and clearly identify the cause of potentially hazardous volcano flank instability and highlight the importance of global flank deformation monitoring campaigns. It is clear that long-term deformation and instabilities are ubiquitous at many volcanoes and may evolve rapidly with the onset or renewal of increased eruptive activity.

### Limitations of the ground deformation analysis

This study of ground deformation from coastal volcanoes in SE Asia reveals a wide range of deformation patterns, magnitudes and rates, highlighting that volcano deformation is a widespread, yet varied, phenomenon, which likely reflects a range of contributing, underlying mechanisms. However, we emphasise that deformation analysis on volcanic islands imparts limitations, raising uncertainties regarding both the quantified values of deformation and the interpretation of the origin of deformation.

One of the challenges faced in our InSAR survey of volcanoes in SE-Asia is presented by the ubiquitous vegetation, lowering the fraction of areas with adequate coherence. This causes poor coverage of deformation maps and limits interpretability and suitable locations for the InSAR reference points that can be considered stable. If possible, the reference points were picked based on differential movement, placing them as close to, but outside detected deformation. But in some cases, due to a lack of distal coherence (Bam and Serua volcanoes), the reference point had to be picked directly adjacent to the summit area, where deformation is most commonly observed at other volcanoes, so our observations of relative displacements to this point and could be larger if this point also deforms. Similarly, for island volcanoes, the reference point was selected close to the shore, though the volcanic edifices extend undersea and are therefore likely to undergo additional deformation extending underwater. As we could not test whether the chosen reference points reflect fully stable ground, we are mindful that our InSAR datasets may not permit the detection of larger-/regional-scale deformation signals, although they allow the identification of relative deformation from the reference with respect to the volcanic edifice. We also cannot verify our deformation data with independent methods (e.g. GNSS data) as the majority of volcanoes investigated herein are sparsely monitored (if at all), which would enable us to correct for reference point deformation. Thus, the displacements reported may generally be regarded as minimum values in most cases. Other geodetic data such as tiltmeters or regular aerial photogrammetry or lidar surveys could further corroborate these measured deformations and strengthen interpretations. Additionally, a broader use of radar wavelength may enhance insights on deformation, specifically the use of L-band sensors such as the ALOS 2 and 4 or the upcoming NISAR could significantly improve the coherence in vegetated areas (e.g. Pritchard et al. 2018), enabling more robust phase unwrapping and reference point selection. In turn, L-band may lose coherence for specific lithologies such as clastic deposit types due to the increased ground penetration depths and the susceptibility of such deposits to volumetric compaction (Arab-Sedze et al. 2014), so it may need multiple sensor types to fully interpret deformation processes at tropical volcanoes.

Another issue for InSAR is presented by atmospheric phase disturbances, which are particularly important in tropical regions such as SE-Asia and can produce apparent deformation in the range of cm/yr (e.g. at Agung volcano; Yip et al. 2019). This has led to past misinterpretations of deformation sources (e.g. at Agung and possibly Merapi; Chaussard et al. 2013). For the data used herein, ERA5 was used to correct for such atmospheric phase delays, which is a widely used global atmospheric model and offers a general improvement on previous versions in terms of temporal (6 h) and spatial (~30 km) resolution (Zhang et al. 2022). Application to our InSAR timeseries successfully removed deformation trends that could be attributed to seasonal atmospheric fluctuations, so our data can be expected to reflect actual ground deformation. Despite this, it is known that even the improved ERA5 resolution may not always fully capture small-scale atmospheric differences around high topographic relief (such as volcanoes; e.g. Albino et al. 2020), hence, we cannot fully rule out some atmospheric impacts for those volcanoes with tall stratocones (e.g. Karangetang or Ulawun). However, since we observe increases in deformation rates at multiple such volcanoes that correlate with eruptive events, we surmise that these deformations do not relate to atmospheric disturbances.

In our analysis, we have attributed changes in observed displacement rates to multiple types of volcanic activity. The eruptive information gathered is limited to written reports and observations of thermal anomalies characterised by the VRP metric and is therefore susceptible to being incomplete. Due to the sparse monitoring of most volcanoes, we anticipate that some events, particularly of smaller magnitude, are missing in the records. Similarly, the accuracy of VRP relies on cloud-free visibility during satellite measurements, but the tropical climate of SE Asia results in frequent cloud coverage during the course of the year. Hence, it is also possible that some eruptive events remain undiscovered.

Our interpretations of the underlying cause of deformation are provided as guidance only as they simply rely on the spatial extent of displacements, their relative rates as well as circumstantial evidence, without a detailed high-resolution survey of the geology and the geophysical signal. Hence, the interpretations should not be considered conclusive but are provided to stimulate new investigations in these key areas. This may help target investigations in these remote locations, which may exhibit an important potential hazard to distal regions.

## Conclusions

Our analysis of 20 volcanoes in Southeast Asia (Indonesia and Papua New Guinea) has revealed a broad spectrum of ground deformation patterns and rates:

1. Eighteen of the 20 studied volcanoes (~90%) show multi-year ground deformation. LOS displacement rates vary greatly on the order of millimetres to decimetres per year with either persistent or episodic deformation periods. We observe predominantly LOS increases (associated with subsidence) that affect the entire edifice or individual flanks.
2. At several volcanoes, the LOS displacement rates were unsteady and found to be affected by increases in volcanic activity, causing increase in displacement rates for months to years (Gamalama, Kadovar, Karangetang, Langila, Manam, Ruang, Ulawun). This highlights a close relationship between volcanic activity and long-term volcano displacement rates.
3. The volcanoes Anak Krakatau, Banda Api, Batu Tara, Iliwerung, Iya, Lewotobi, Nila, Sangean Api, Serua and Sirung were found to deform at steady (linear) displacement rates, implying some deformations to be stable in time and space, independent of volcanic activity.
4. Deformation appears primarily gravity-driven rather than by magmatic processes and includes flank instability by flank creep, deep-seated faulting, mechanical compaction of volcaniclastic materials, but also non-gravitational processes such as thermal cooling contraction. The horizontal displacements may help identify or discriminate between the key processes via an assessment of the overall ground displacements (e.g. convergent vs divergent vs unilateral horizontal deformation of volcanic edifice).
5. We identify ongoing flank instability at Anak Krakatau (in the same location as the previous collapse). Similarly, likely flank instability is found at Lewotobi (Laki-Laki), Sirung and Ulawun, all exhibiting significant downslope deformation, posing potential collapse hazards. Tentative evidence for flank instability was found at Banda Api, Kadovar, Karangetang, Manam, Nila and Ruang.

We call for increased long-term monitoring of volcano deformation worldwide as it is clear that volcanic activity can rapidly change deformation rates and the stability of volcano flanks for many years, but the mechanisms remain poorly understood. Our results showcase that most volcanoes experience long-term deformation, necessitating systematic monitoring to identify potentially hazardous instability. In areas with poor InSAR coherence, additional geodetic monitoring (e.g. GNSS, tilt sensors or photogrammetry) could greatly improve signal detection and interpretation, enabling better early-warning capabilities.

## Supplementary information

Below is the link to the electronic supplementary material.

Supplementary file 1 (PDF 8.37 MB)

Supplementary file 2 (ZIP 69.6 MB)

## Data Availability

The processed InSAR data files containing the point timeseries (as shapefiles built with Mintpy) and the temporal coherence maps (as geotiffs) for each volcano can be downloaded from the supplementary files to this article. A list with a data overview containing volcano names, observation time period, orbit information and reference point locations is also provided with the files.
