# Supplementary material for: Long-term deformation of coastal volcanoes in SE-Asia: linking displacement rates, volcanic activity and flank instabilities
Source: Bull Volcanol. 2025 Dec 13;88(1):4. doi: 10.1007/s00445-025-01915-z (PMC12700928; doi:10.1007/s00445-025-01915-z)
Supplement: Supplementary file 1 — (PDF 8.37 MB) [file 445_2025_1915_MOESM1_ESM.pdf]

Supplementary Material:
Long-term deformation of coastal volcanoes in SE-Asia:
Linking displacement rates, volcanic activity and flank instabilities

Edgar U. Zorn, Falk Amelung, Francesco Massimetti, Marco Laiolo, Diego Coppola, Thomas R. Walter, Yan Lavallée, Herlan Darmawan

Anak Krakatau Ascending Track 171 (May 2021 - Aug 2024)

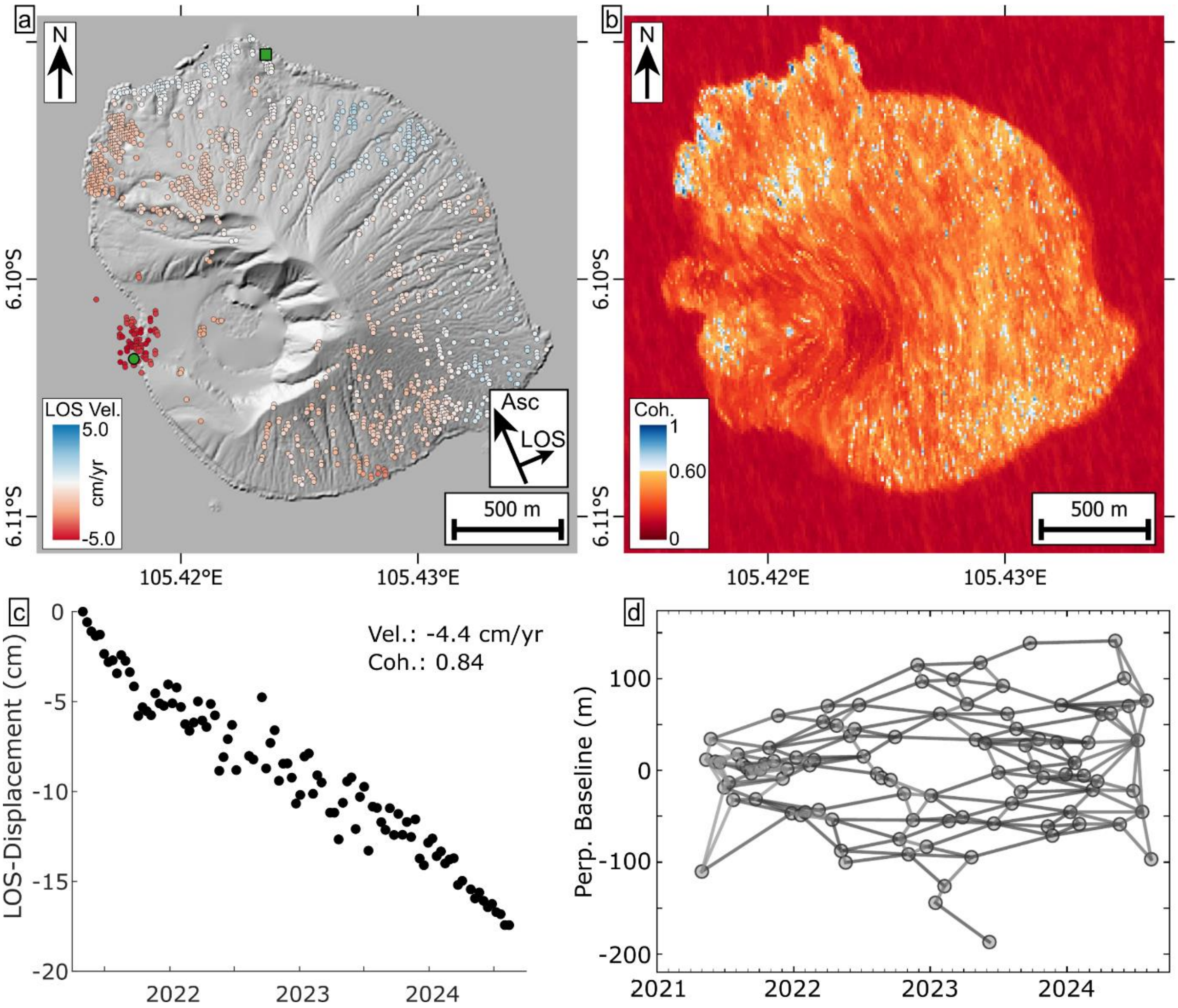

Figure S1: LOS-Timeseries InSAR data for Anak Krakatau volcano, Indonesia, in ascending orbit showing (a) the LOS-velocity map on a hillshade background. The green square marks the reference point and the green circle marks the selected timeseries point plotted in (c), (b) the temporal coherence map, (c) the selected point time series with the average velocity and the coherence for the selected point, and (d) the interferogram connections network plot.

Anak Krakatau Descending Track 87 (May 2021 - Jun 2023)

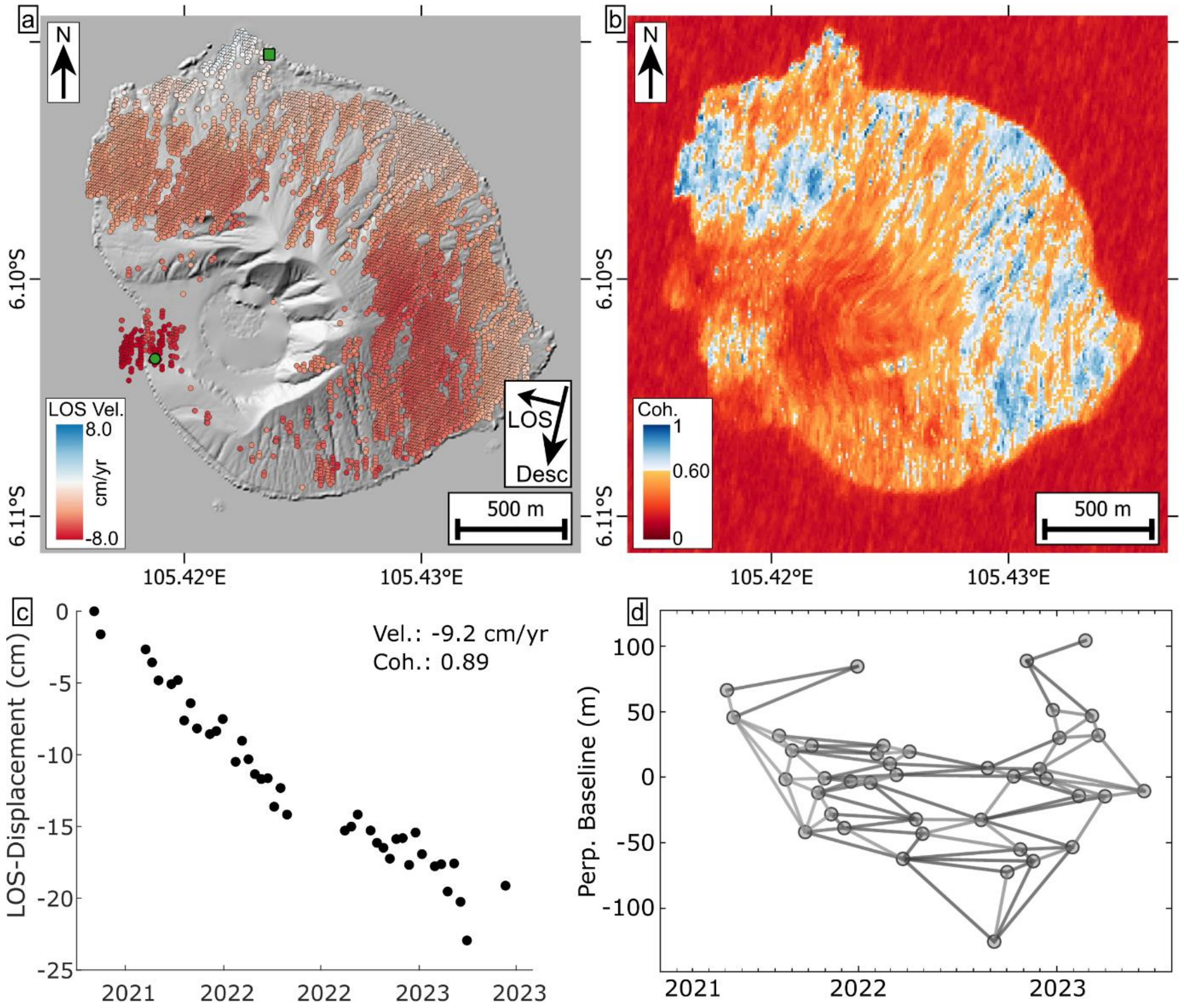

Figure S2: LOS-Timeseries InSAR data for Anak Krakatau volcano, Indonesia, in descending orbit showing (a) the LOS-velocity map on a hillshade background. The green square marks the reference point and the green circle marks the selected timeseries point plotted in (c), (b) the temporal coherence map, (c) the selected point time series with the average velocity and the coherence for the selected point, and (d) the interferogram connections network plot.

Awu Descending Track 163 (Jan 2020 - Aug 2024)

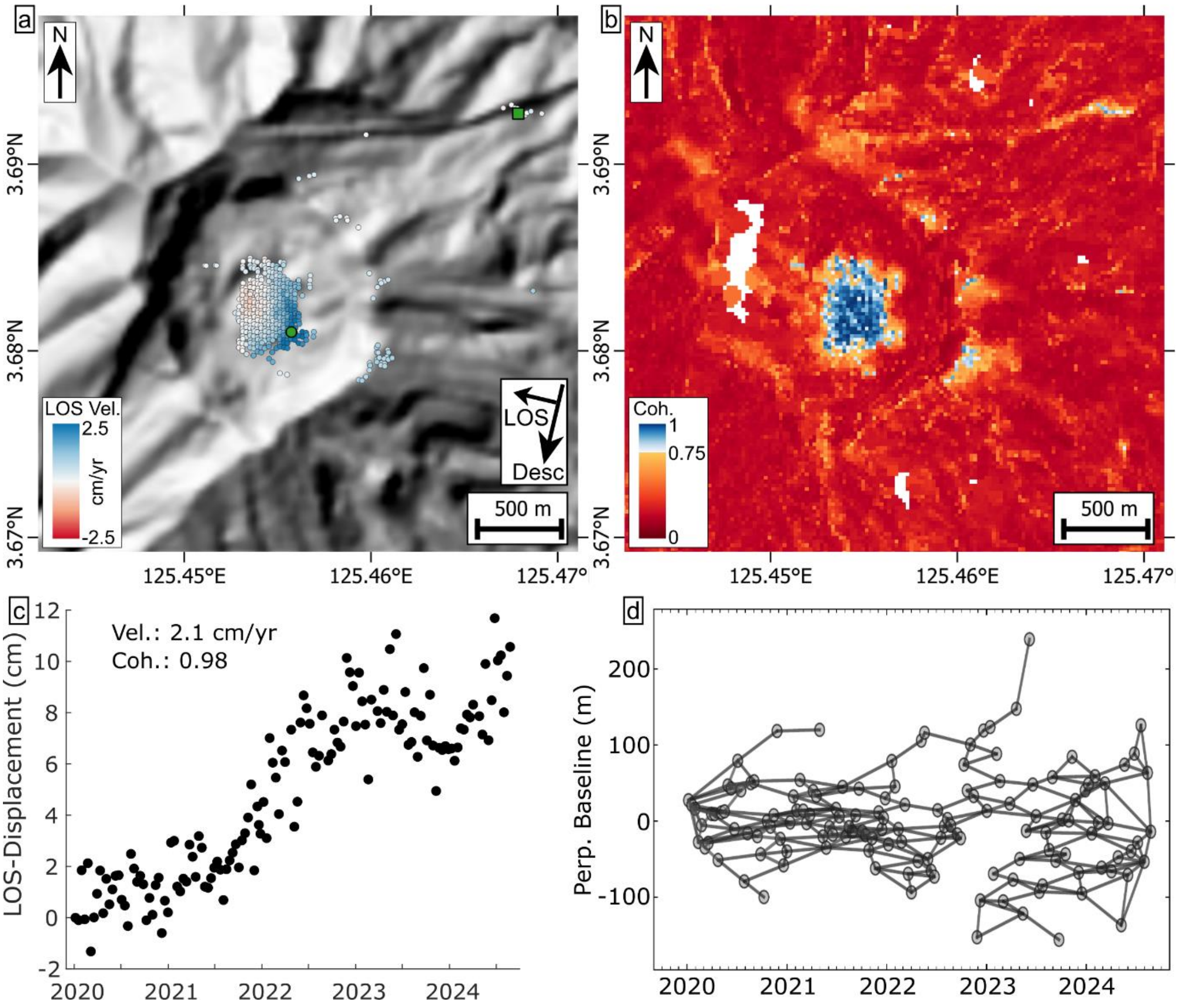

Figure S3: LOS-Timeseries InSAR data for Awu volcano, Indonesia, in descending orbit showing (a) the LOS-velocity map on a hillshade background. The green square marks the reference point and the green circle marks the selected timeseries point plotted in (c), (b) the temporal coherence map, (c) the selected point time series with the average velocity and the coherence for the selected point, and (d) the interferogram connections network plot.

The coastal volcano last erupted in 2004, but periodic unrest due to seismicity persists since July 2003. Almost the entire edifice is covered by dense vegetation, leaving almost no coherent areas except within the crater. Here, the InSAR suggests a LOS-inflation on the inner eastern crater side (at ~2.1 cm/yr), whereas the crater center shows a minor LOS-subsidence. This likely indicates a poor reference location, as it is unlikely that both directionalities occur within such a short ~200 m area. Some deformation is clearly deformation occurring at the crater of Awu, however, due to the limited coherent areas the directionality cannot be reliably established, preventing any meaningful interpretation of the signal.

Bam Descending Track 133 (Jan 2017 - Jul 2024)

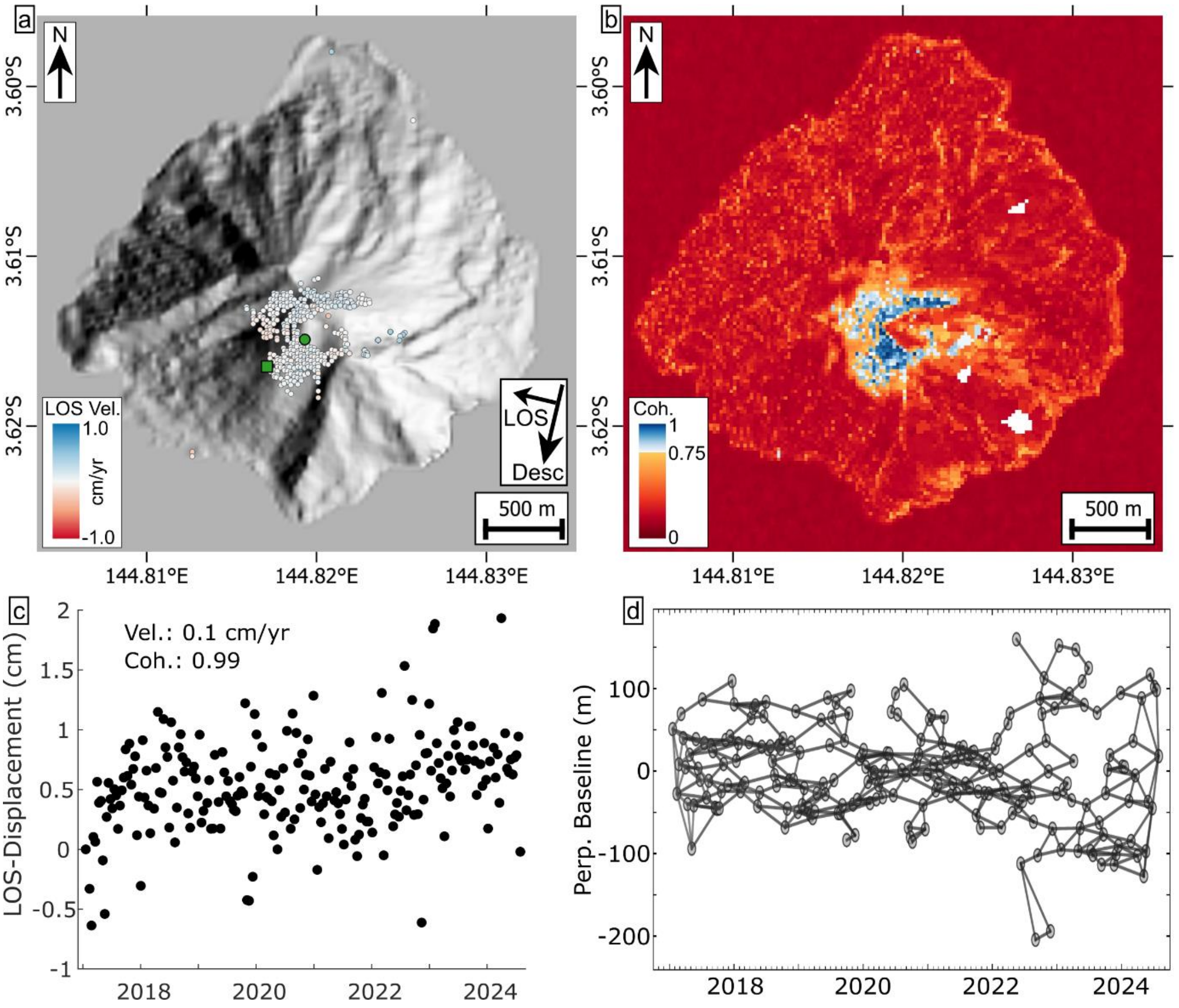

Figure S4: LOS-Timeseries InSAR data for Bam volcano, Papua New Guinea, in descending orbit showing (a) the LOS-velocity map on a hillshade background. The green square marks the reference point and the green circle marks the selected timeseries point plotted in (c), (b) the temporal coherence map, (c) the selected point time series with the average velocity and the coherence for the selected point, and (d) the interferogram connections network plot.

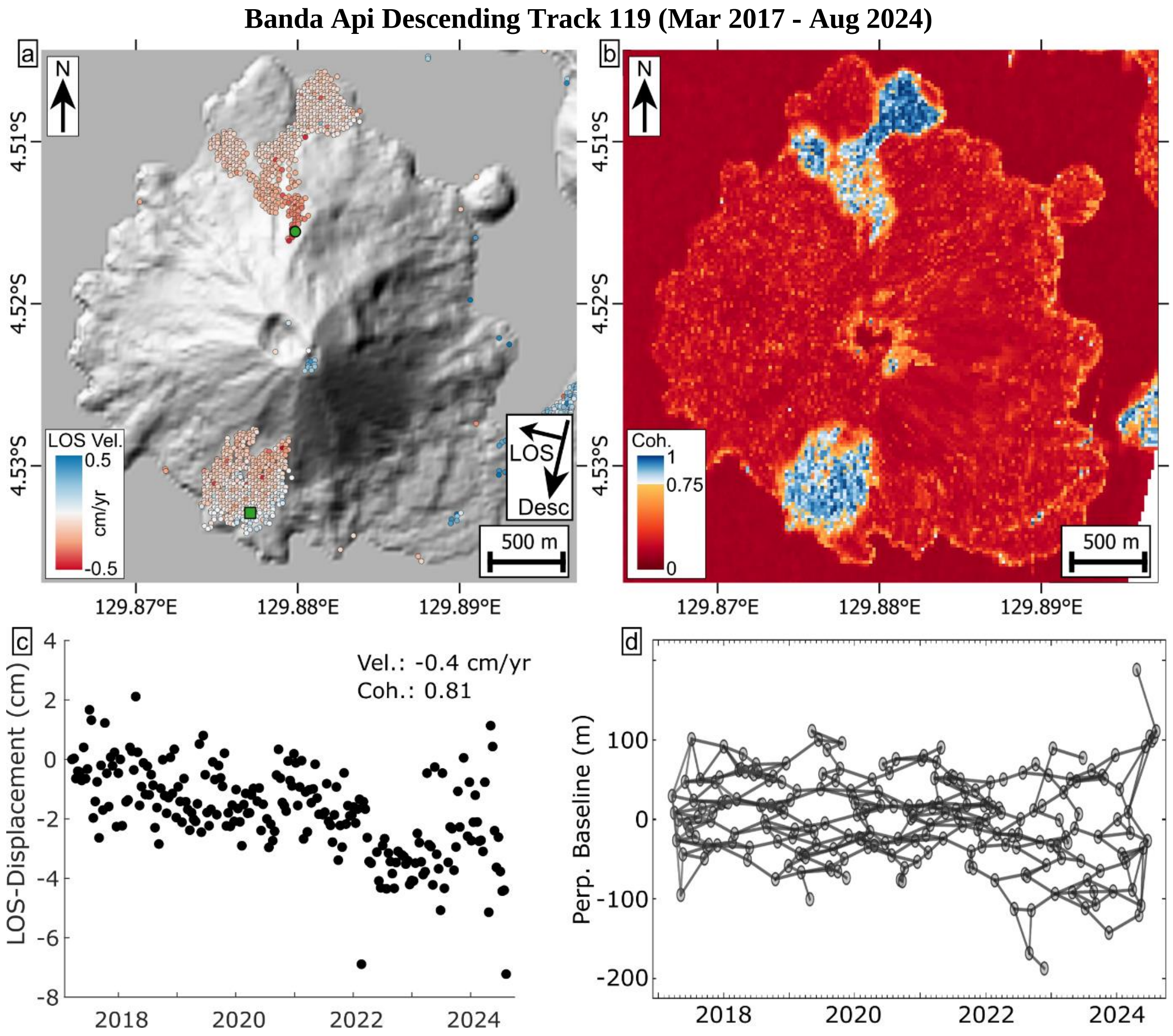

Figure S5: LOS-Timeseries InSAR data for Banda Api volcano, Indonesia, in descending orbit showing (a) the LOS-velocity map on a hillshade background. The green square marks the reference point and the green circle marks the selected timeseries point plotted in (c), (b) the temporal coherence map, (c) the selected point time series with the average velocity and the coherence for the selected point, and (d) the interferogram connections network plot.

Batu Tara Ascending Track 39 (Nov 2016 - Jan 2024)

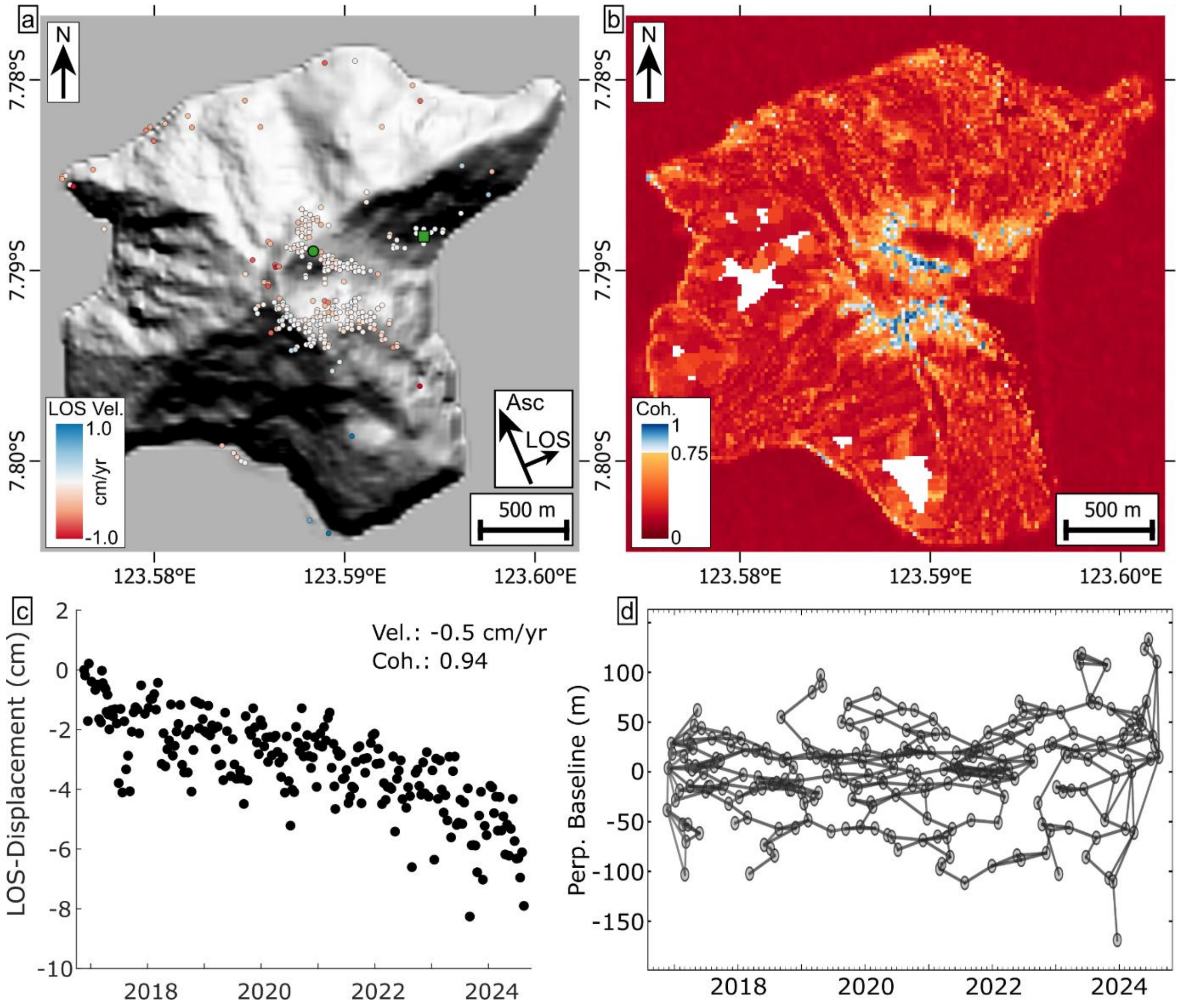

Figure S6: LOS-Timeseries InSAR data for Batu Tara volcano, Indonesia, in ascending orbit showing (a) the LOS-velocity map on a hillshade background. The green square marks the reference point and the green circle marks the selected timeseries point plotted in (c), (b) the temporal coherence map, (c) the selected point time series with the average velocity and the coherence for the selected point, and (d) the interferogram connections network plot.

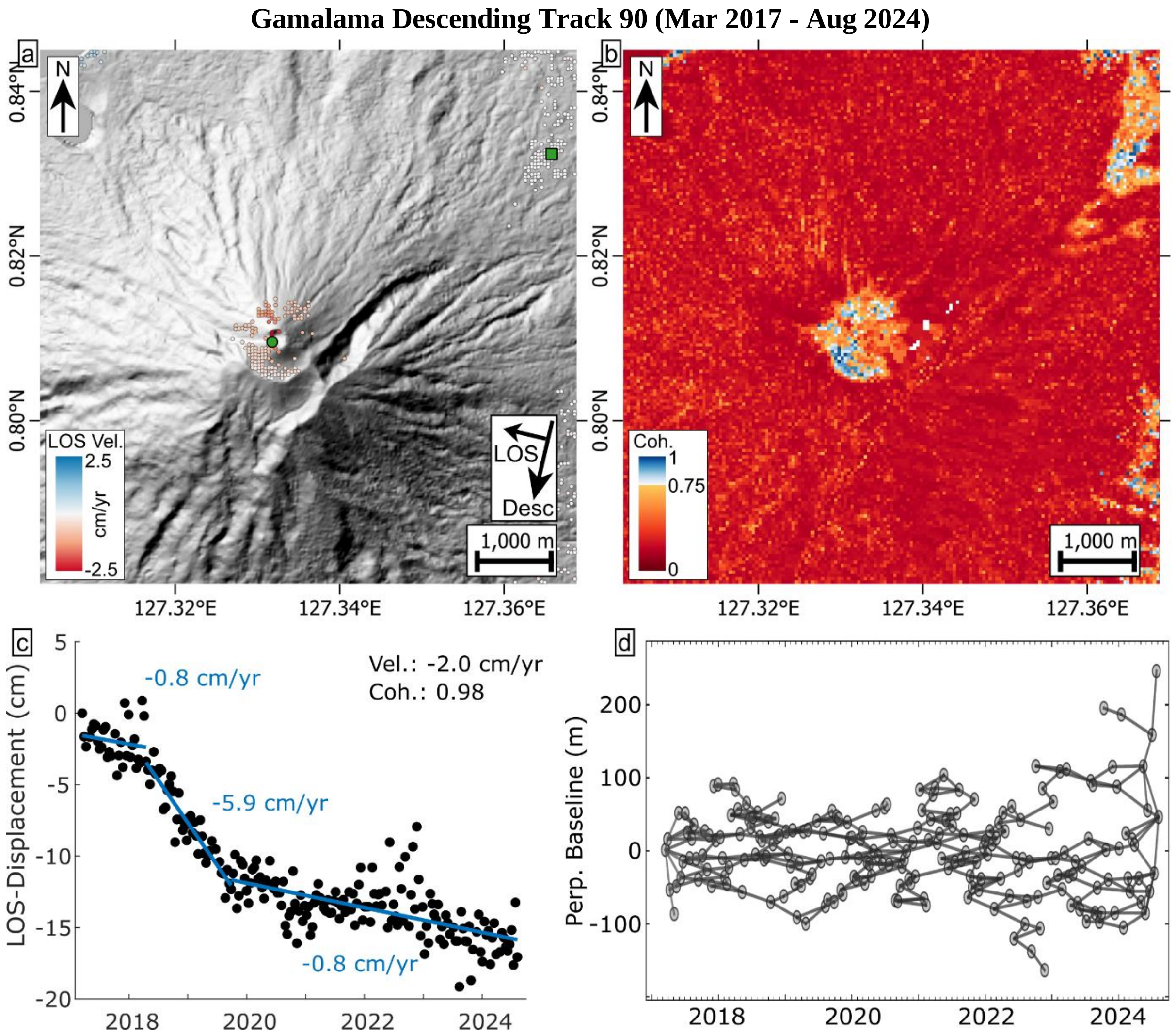

Figure S7: LOS-Timeseries InSAR data for Gamalama volcano, Indonesia, in descending orbit showing (a) the LOS-velocity map on a hillshade background. The green square marks the reference point and the green circle marks the selected timeseries point plotted in (c), (b) the temporal coherence map, (c) the selected point time series with the average velocity and the coherence for the selected point, and (d) the interferogram connections network plot.

Iliwerung Ascending Track 39 (Jan 2017 - Mar 2024)

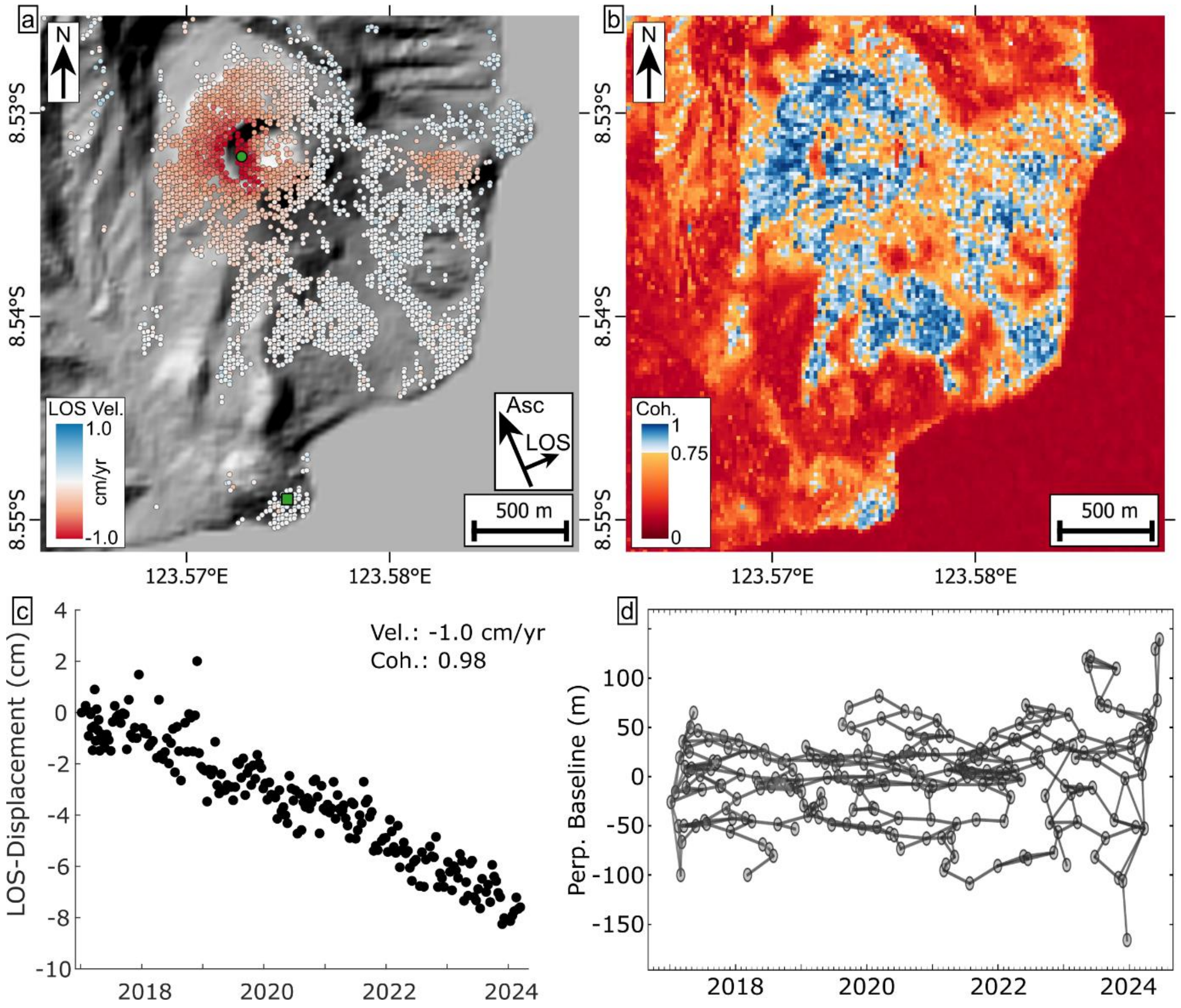

Figure S8: LOS-Timeseries InSAR data for Iliwerung volcano, Indonesia, in ascending orbit showing (a) the LOS-velocity map on a hillshade background. The green square marks the reference point and the green circle marks the selected timeseries point plotted in (c), (b) the temporal coherence map, (c) the selected point time series with the average velocity and the coherence for the selected point, and (d) the interferogram connections network plot.

Iliwerung Descending Track 90 (Jan 2017 - Jul 2024)

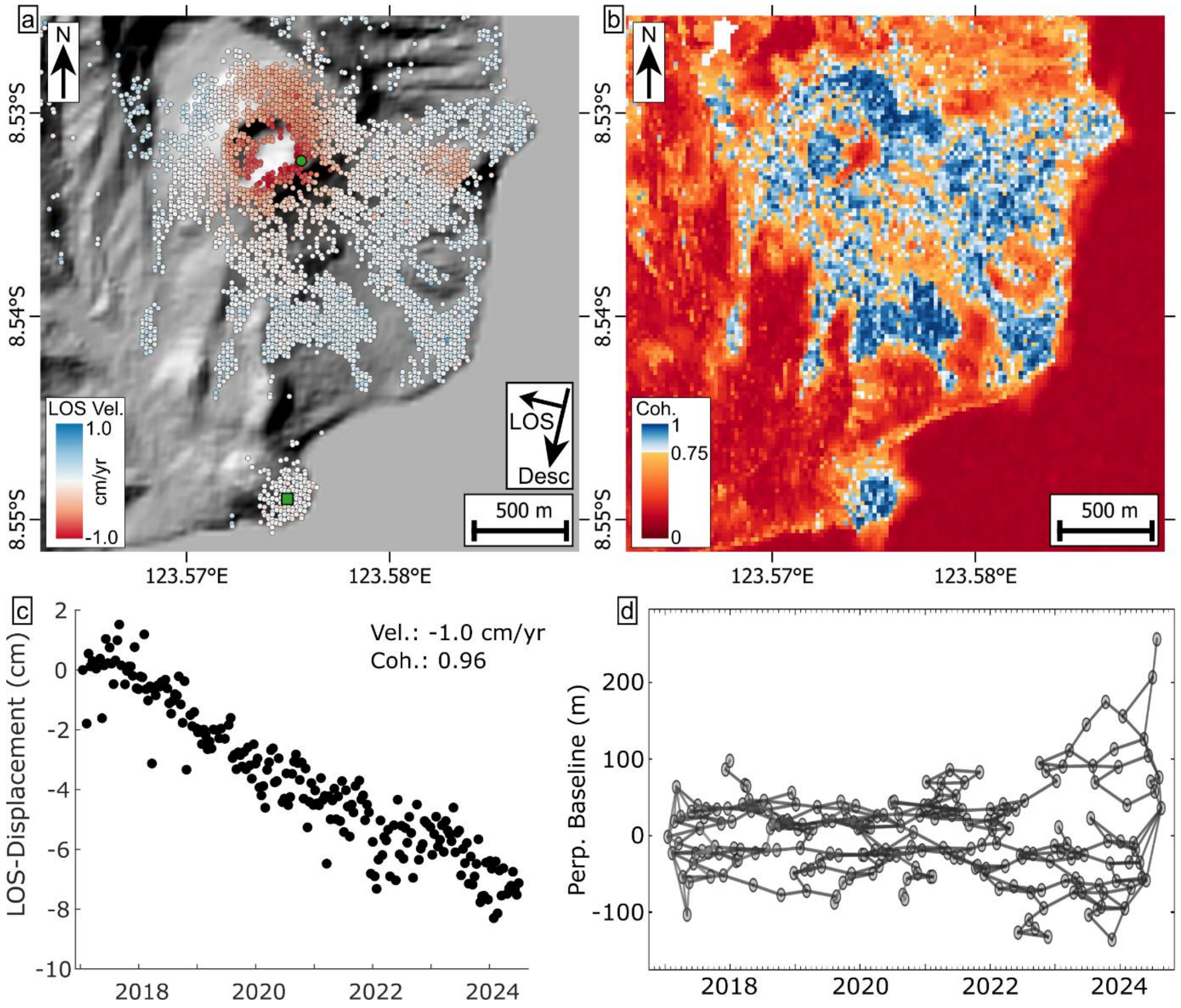

Figure S9: LOS-Timeseries InSAR data for Iliwerung volcano, Indonesia, in descending orbit showing (a) the LOS-velocity map on a hillshade background. The green square marks the reference point and the green circle marks the selected timeseries point plotted in (c), (b) the temporal coherence map, (c) the selected point time series with the average velocity and the coherence for the selected point, and (d) the interferogram connections network plot.

Iya Descending Track 163 (Jan 2017 - Nov 2024)

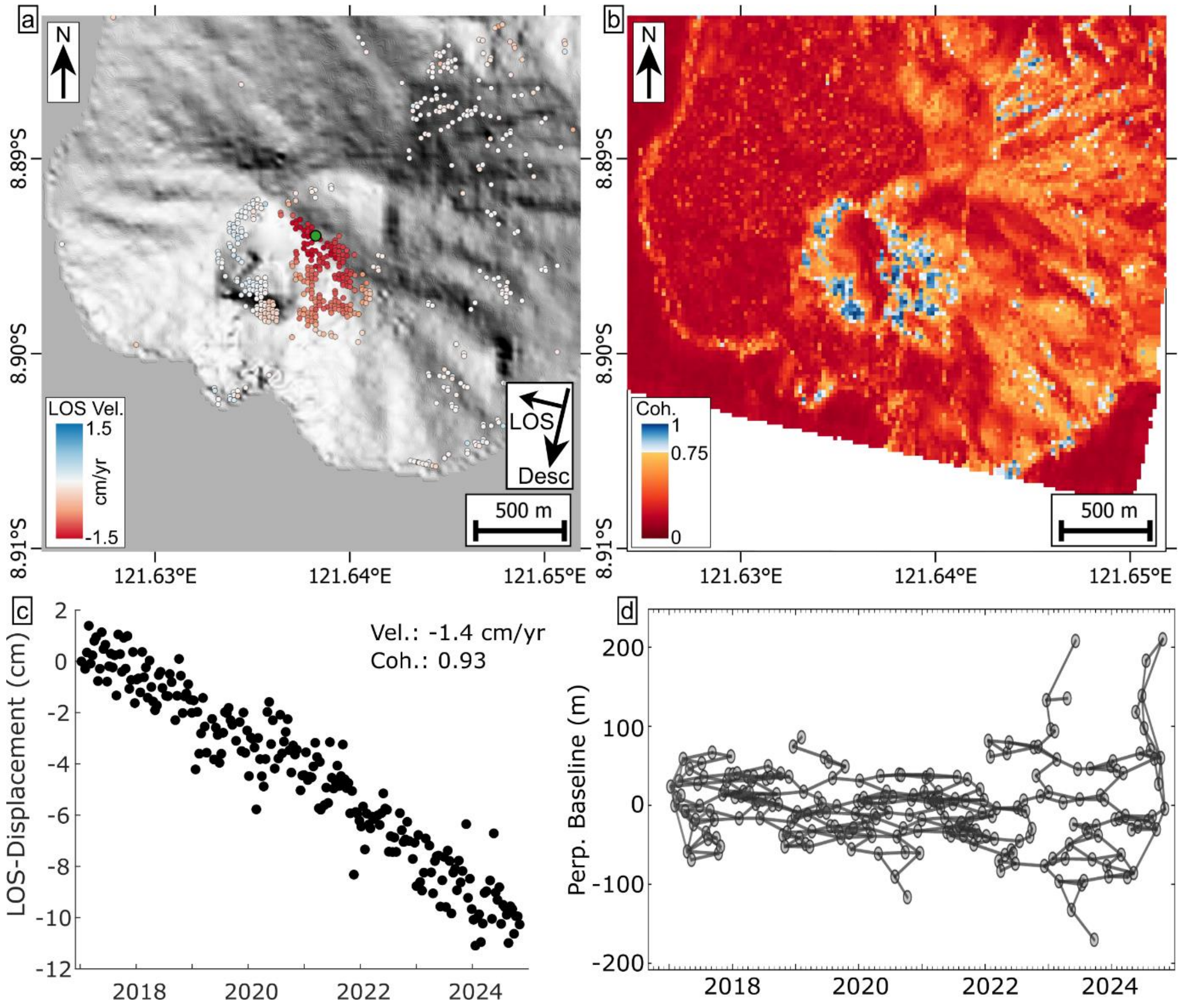

Figure S10: LOS-Timeseries InSAR data for Iya volcano, Indonesia, in descending orbit showing (a) the LOS-velocity map on a hillshade background. The green square marks the reference point and the green circle marks the selected timeseries point plotted in (c), (b) the temporal coherence map, (c) the selected point time series with the average velocity and the coherence for the selected point, and (d) the interferogram connections network plot.

Kadovar Descending Track 133 (Jan 2021 - May 2023)

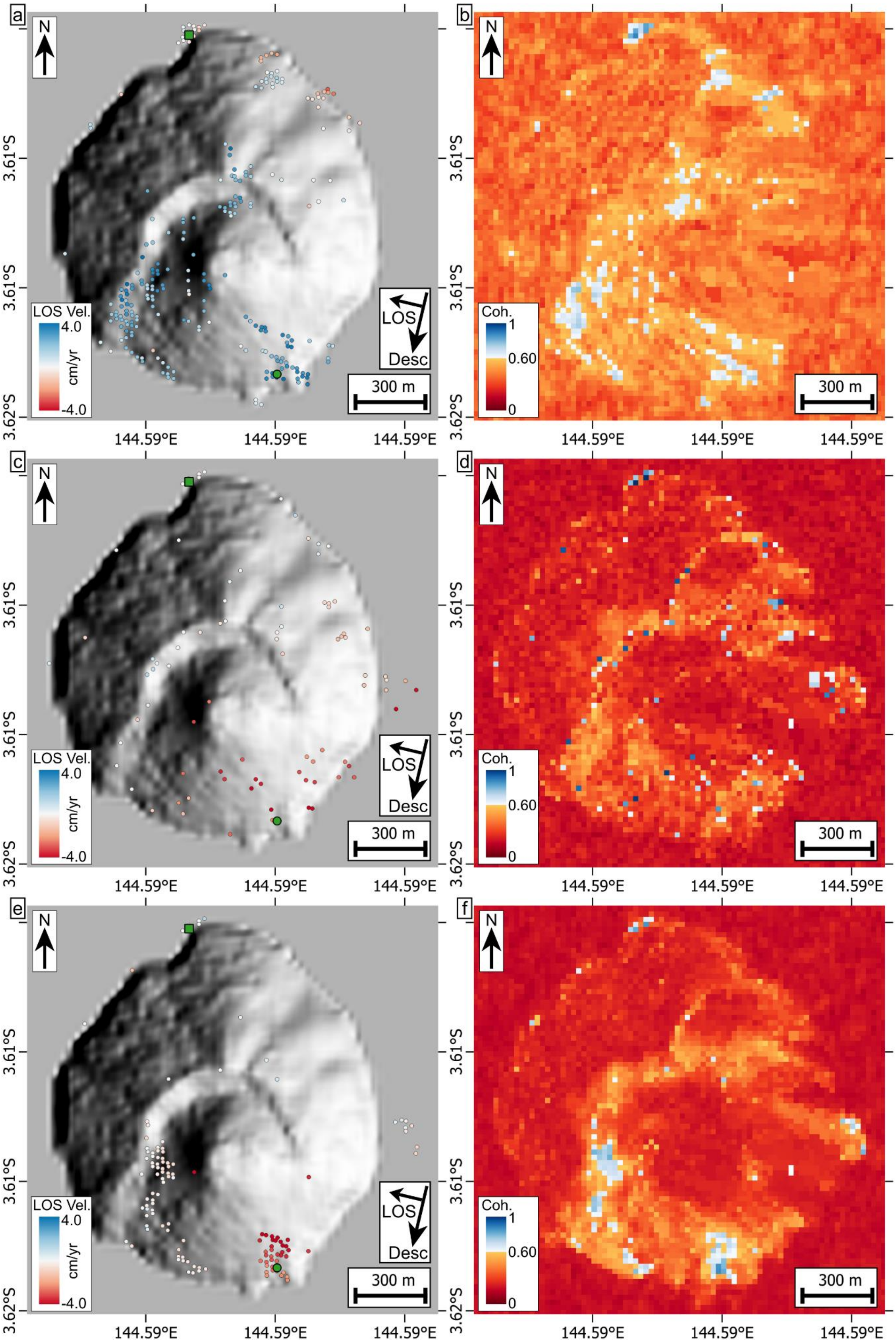

Figure S11: LOS-Timeseries InSAR data for Kadovar volcano (continued below).

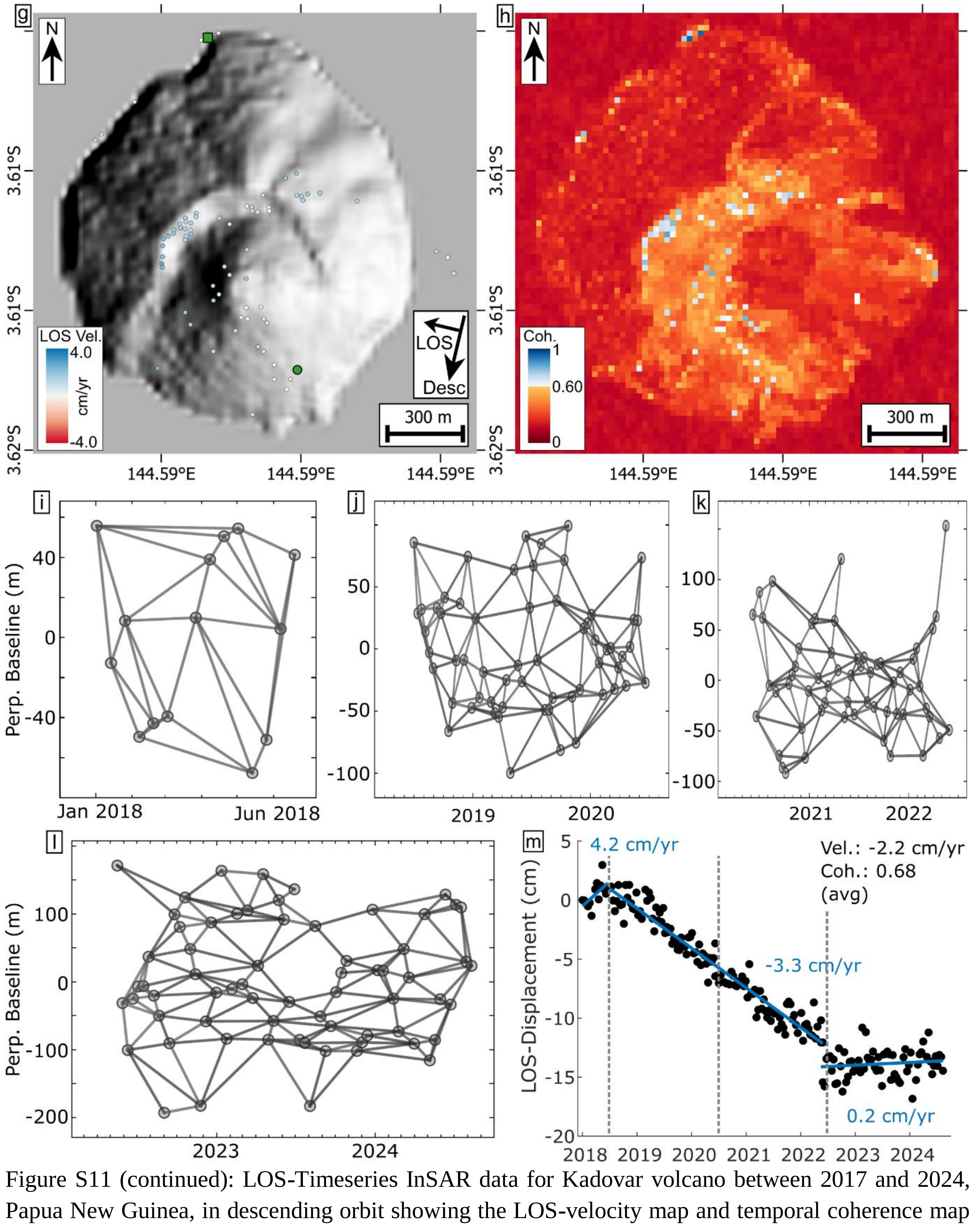

Figure S11 (continued): LOS-Timeseries InSAR data for Kadovar volcano between 2017 and 2024, Papua New Guinea, in descending orbit showing the LOS-velocity map and temporal coherence map for (a,b) January 2018 to June 2018, (c,d) June 2018 to June 2020, (e,f) June 2020 to June 2022 and (g,h) May 2022 to August 2024. The green square marks the reference point and the green circle marks the selected timeseries point plotted in (m) with the respective intervals marked by grey dashed lines in. (i-l) show the interferogram connections network plot for the respective time intervals.

Karangetang Descending Track 163 (Jan 2017 - Jun 2024)

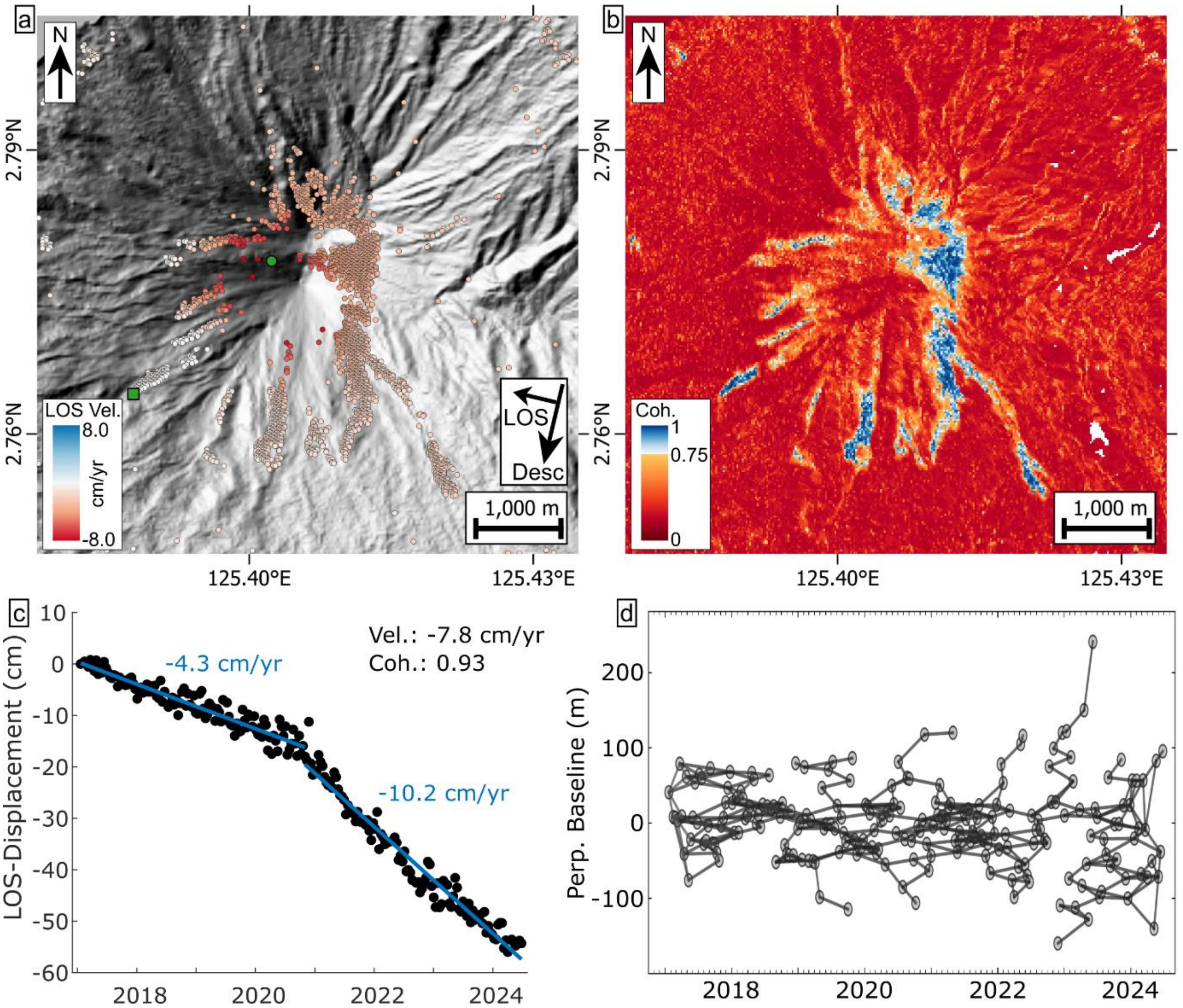

Figure S12: LOS-Timeseries InSAR data for Karangetang volcano, Indonesia, in descending orbit showing (a) the LOS-velocity map on a hillshade background. The green square marks the reference point and the green circle marks the selected timeseries point plotted in (c), (b) the temporal coherence map, (c) the selected point time series with the average velocity and the coherence for the selected point, and (d) the interferogram connections network plot.

Langila Ascending Track 38 (Jan 2016 - Aug 2024)

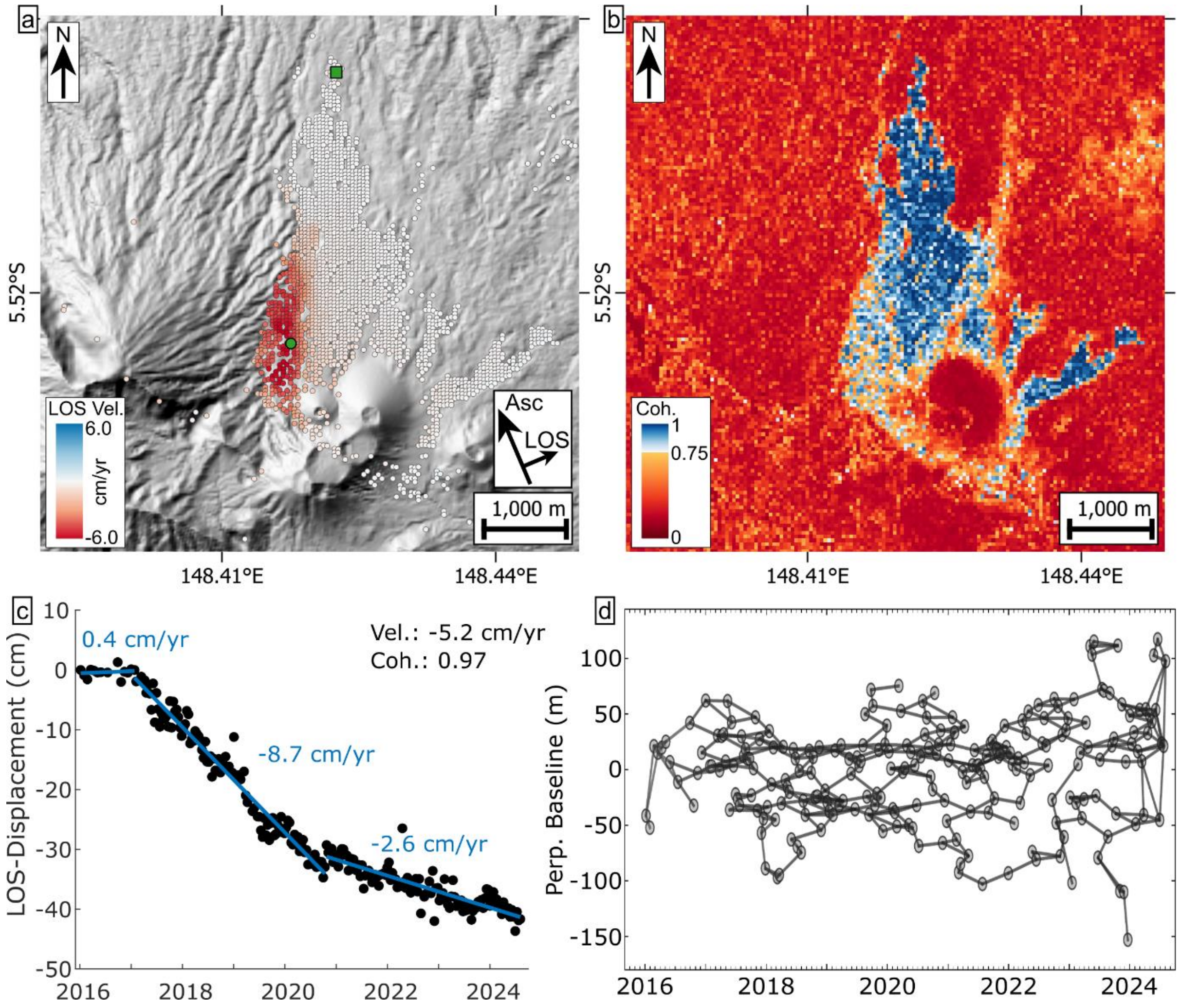

Figure S13: LOS-Timeseries InSAR data for Langila volcano, Papua New Guinea, in ascending orbit showing (a) the LOS-velocity map on a hillshade background. The green square marks the reference point and the green circle marks the selected timeseries point plotted in (c), (b) the temporal coherence map, (c) the selected point time series with the average velocity and the coherence for the selected point, and (d) the interferogram connections network plot.

Langila Descending Track 162 (Nov 2015 - Jul 2024)

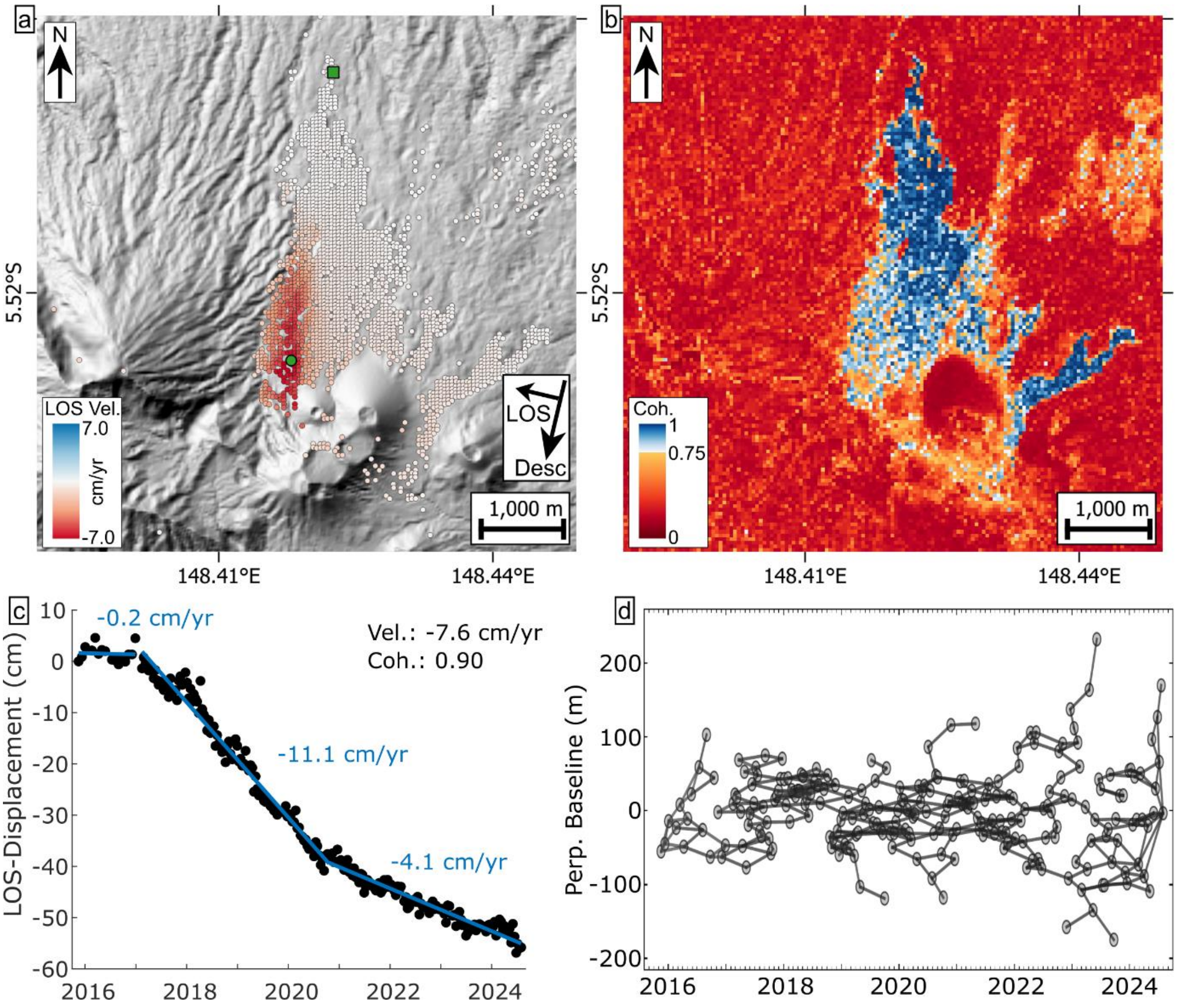

Figure S14: LOS-Timeseries InSAR data for Langila volcano, Papua New Guinea, in descending orbit showing (a) the LOS-velocity map on a hillshade background. The green square marks the reference point and the green circle marks the selected timeseries point plotted in (c), (b) the temporal coherence map, (c) the selected point time series with the average velocity and the coherence for the selected point, and (d) the interferogram connections network plot.

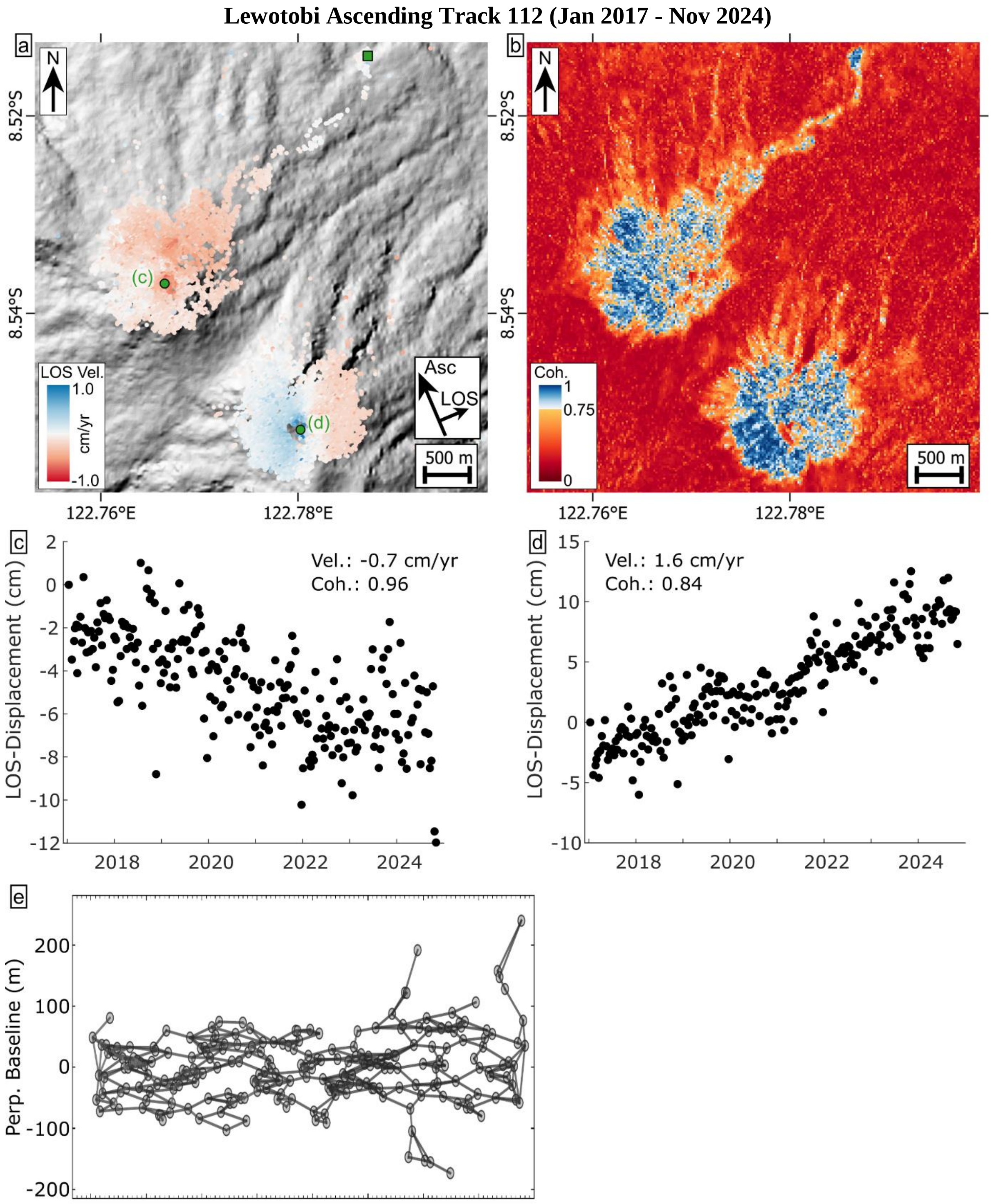

Figure S15: LOS-Timeseries InSAR data for Lewotobi volcano, Indonesia, in ascending orbit showing (a) the LOS-velocity map on a hillshade background. The green square marks the reference point and the two green circles mark the selected timeseries points plotted in (c) and (d). (b) shows the temporal coherence map, (c) the selected point time series on the northern edifice (Laki-Laki) flank, (d) the selected point time series on the southern edifice (Perempuam). All points also report the average velocity and the coherence for the selected points. (e) shows the interferogram connections network plot.

Lewotobi Descending Track Track 163 (Jan 2017 - Nov 2024)

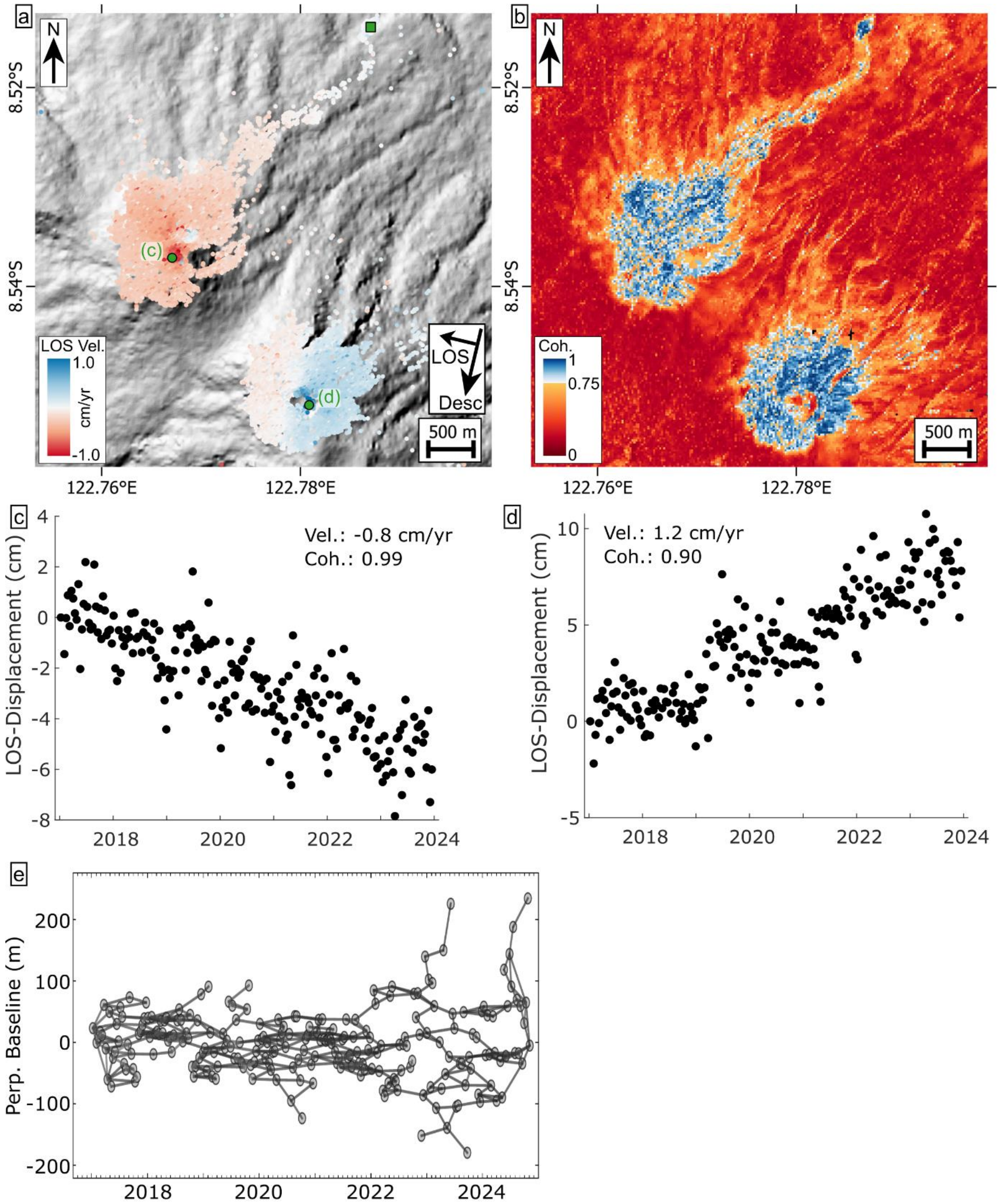

Figure S16: LOS-Timeseries InSAR data for Lewotobi volcano, Indonesia, in descending orbit showing (a) the LOS-velocity map on a hillshade background. The green square marks the reference point and the two green circles mark the selected timeseries points plotted in (c) and (d). (b) shows the temporal coherence map, (c) the selected point time series on the northern edifice (Laki-Laki) flank, (d) the selected point time series on the southern edifice (Perempuam). All points also report the average velocity and the coherence for the selected points. (e) shows the interferogram connections network plot.

Manam Descending Track 60 (Mar 2017 - Jun 2024)

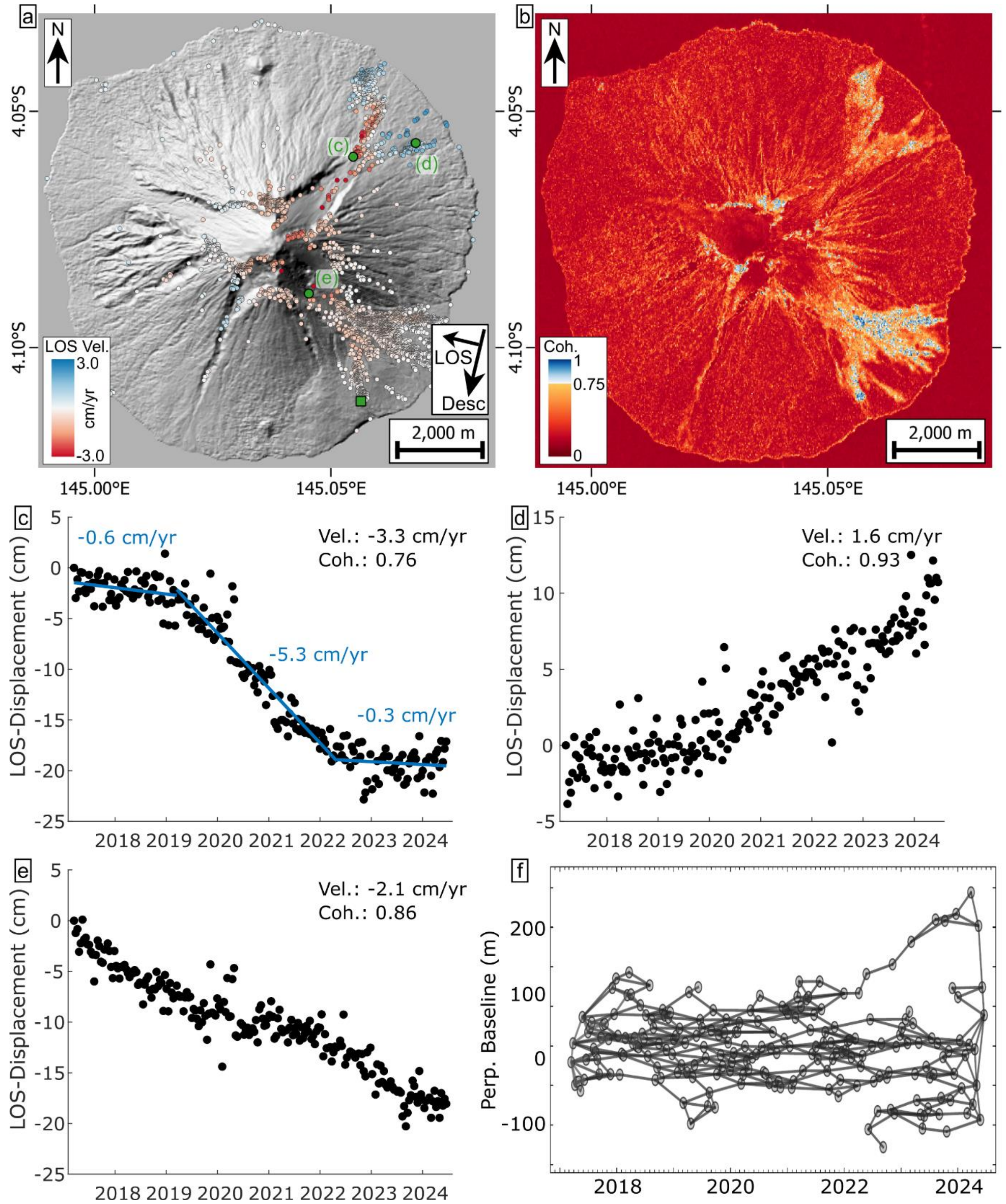

Figure S17: LOS-Timeseries InSAR data for Manam volcano, Papua New Guinea, in descending orbit showing (a) the LOS-velocity map on a hillshade background. The green square marks the reference point and the three green circles mark the selected timeseries points plotted in (c), (d), and (e). (b) shows the temporal coherence map, (c) the selected point time series on the northeast flank, (d) the selected point time series on the northeast shore, and (d) the selected point time series on the southeast flank. All points also report the average velocity and the coherence for the selected points. (f) shows the interferogram connections network plot.

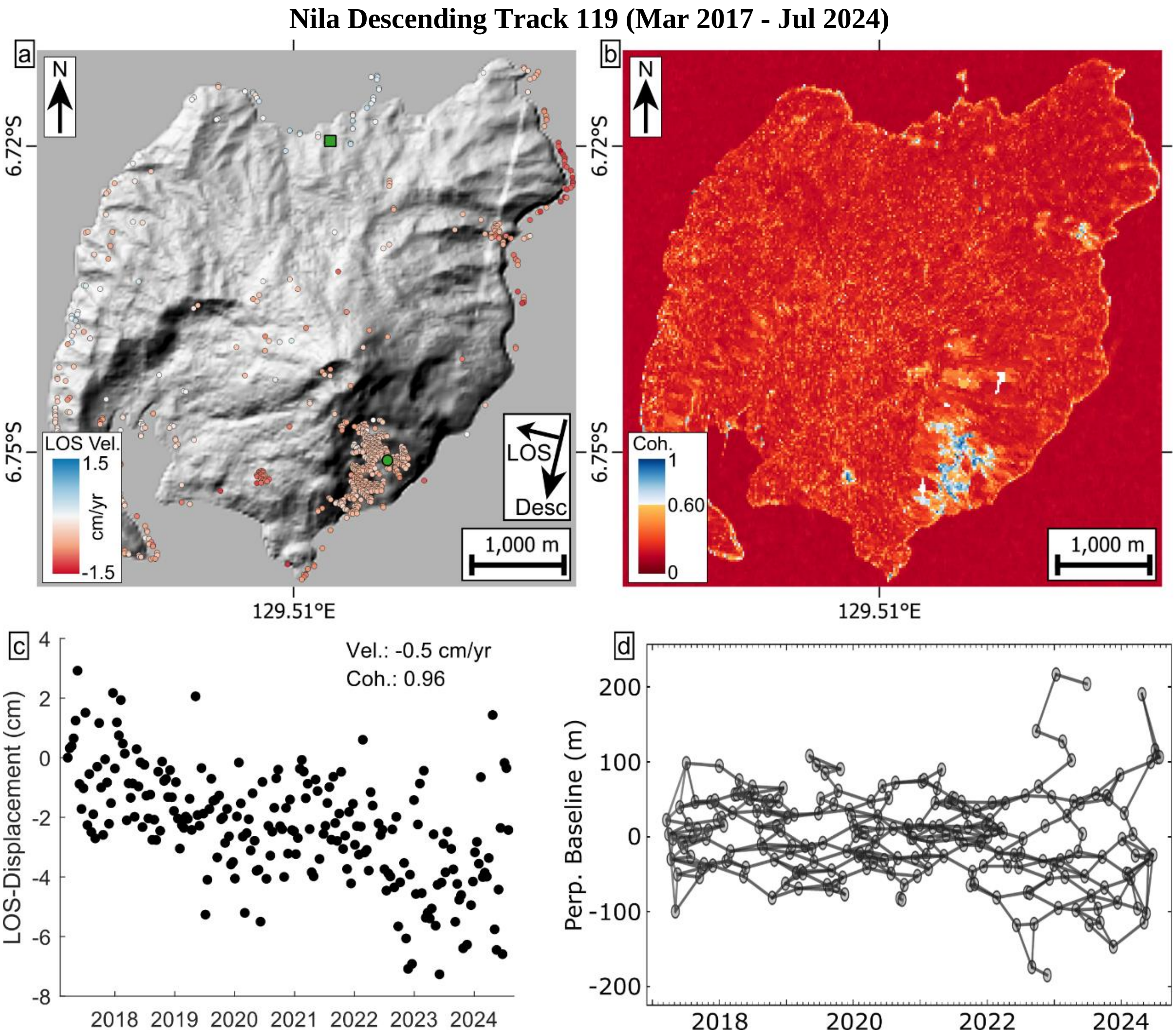

Figure S18: LOS-Timeseries InSAR data for Nila volcano, Indonesia, in descending orbit showing (a) the LOS-velocity map on a hillshade background. The green square marks the reference point and the green circle marks the selected timeseries point plotted in (c), (b) the temporal coherence map, (c) the selected point time series with the average velocity and the coherence for the selected point, and (d) the interferogram connections network plot.

Ruang (Pre-2024 Eruption) Descending Track 163 (Jan 2017 - Apr 2024)

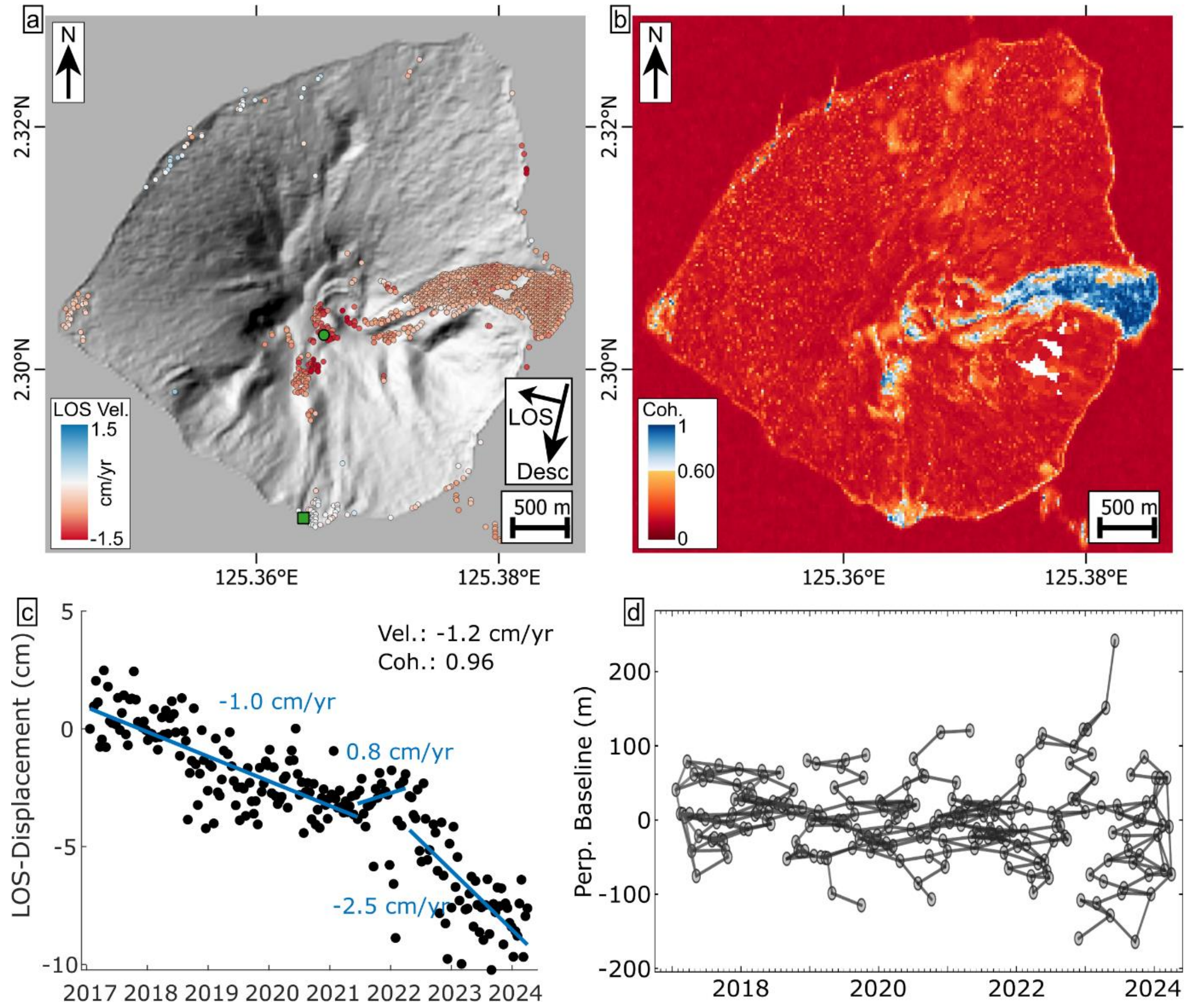

Figure S19: LOS-Timeseries InSAR data for Ruang volcano, Indonesia, between 2017 and 2024 in descending orbit showing (a) the LOS-velocity map on a hillshade background. The green square marks the reference point and the green circle marks the selected timeseries point plotted in (c), (b) the temporal coherence map, (c) the selected point time series with the average velocity and the coherence for the selected point, and (d) the interferogram connections network plot.

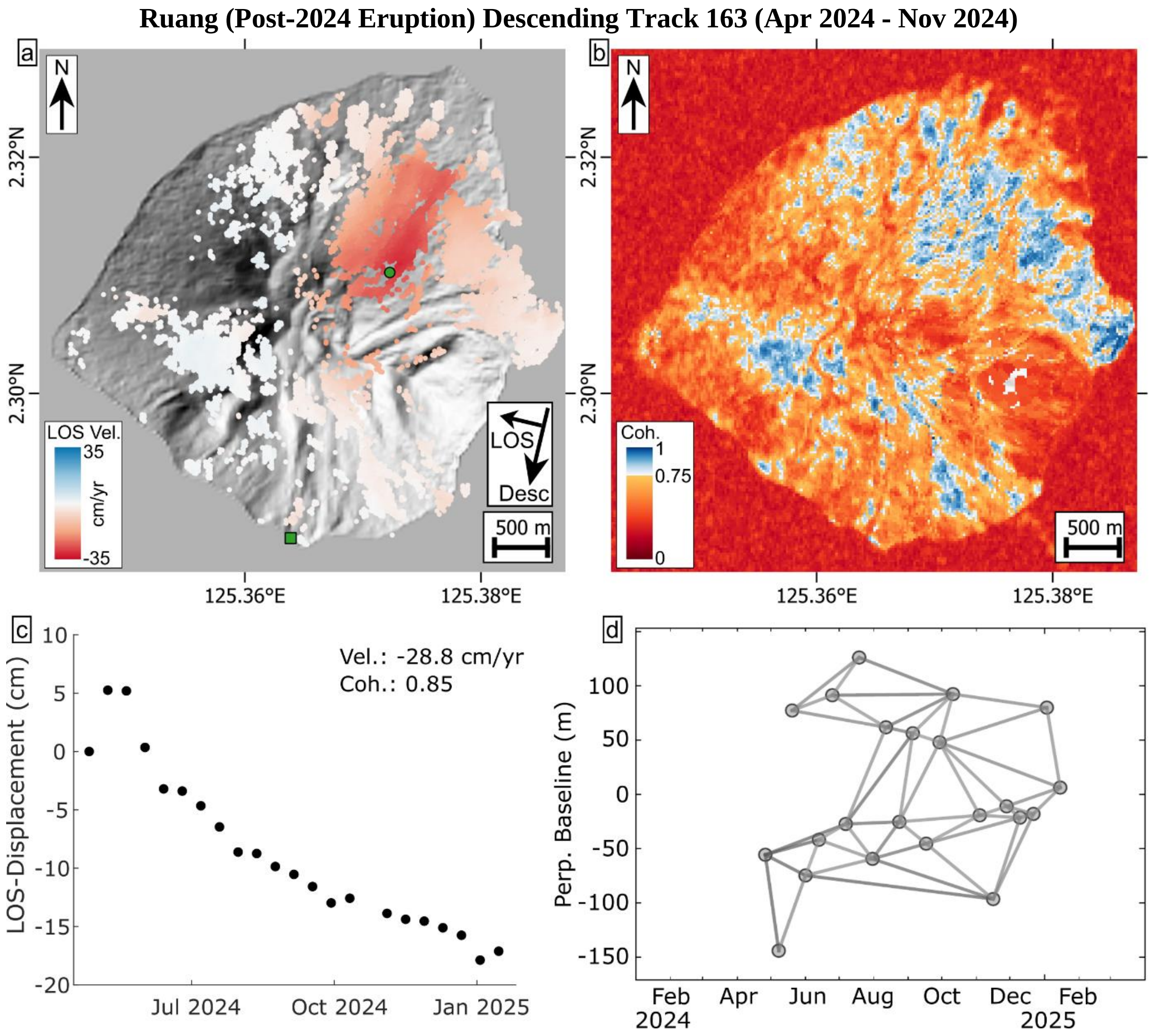

Figure 20: LOS-Timeseries InSAR data for Ruang volcano, Indonesia, after the eruption in April 2024 in descending orbit showing (a) the LOS-velocity map on a hillshade background. The green square marks the reference point and the green circle marks the selected timeseries point plotted in (c), (b) the temporal coherence map, (c) the selected point time series with the average velocity and the coherence for the selected point, and (d) the interferogram connections network plot.

Sangeang Api Descending Track 134 (May 2020 - Aug 2024)

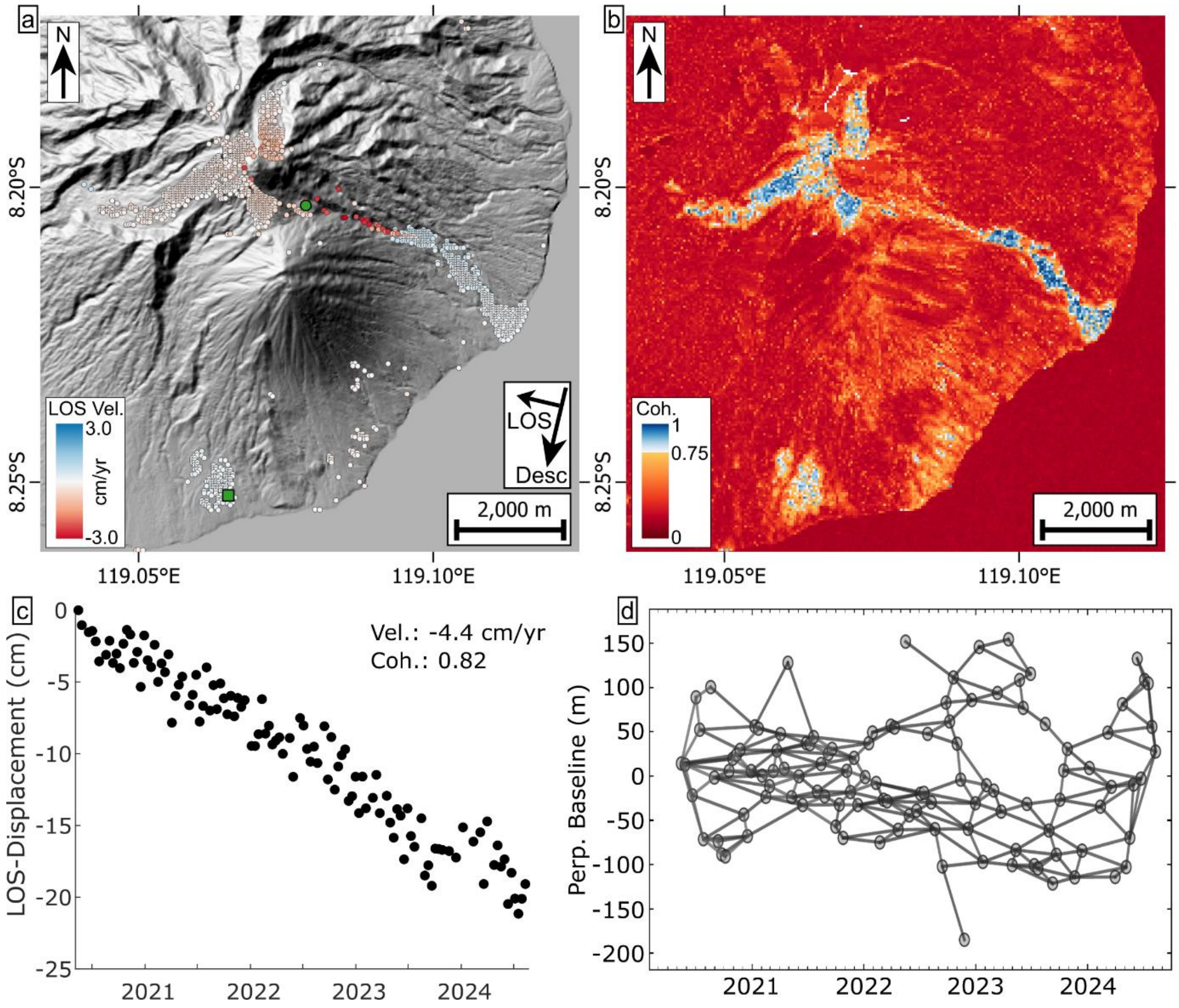

Figure S21: LOS-Timeseries InSAR data for Sangeang Api volcano, Indonesia, in descending orbit showing (a) the LOS-velocity map on a hillshade background. The green square marks the reference point and the green circle marks the selected timeseries point plotted in (c), (b) the temporal coherence map, (c) the selected point time series with the average velocity and the coherence for the selected point, and (d) the interferogram connections network plot.

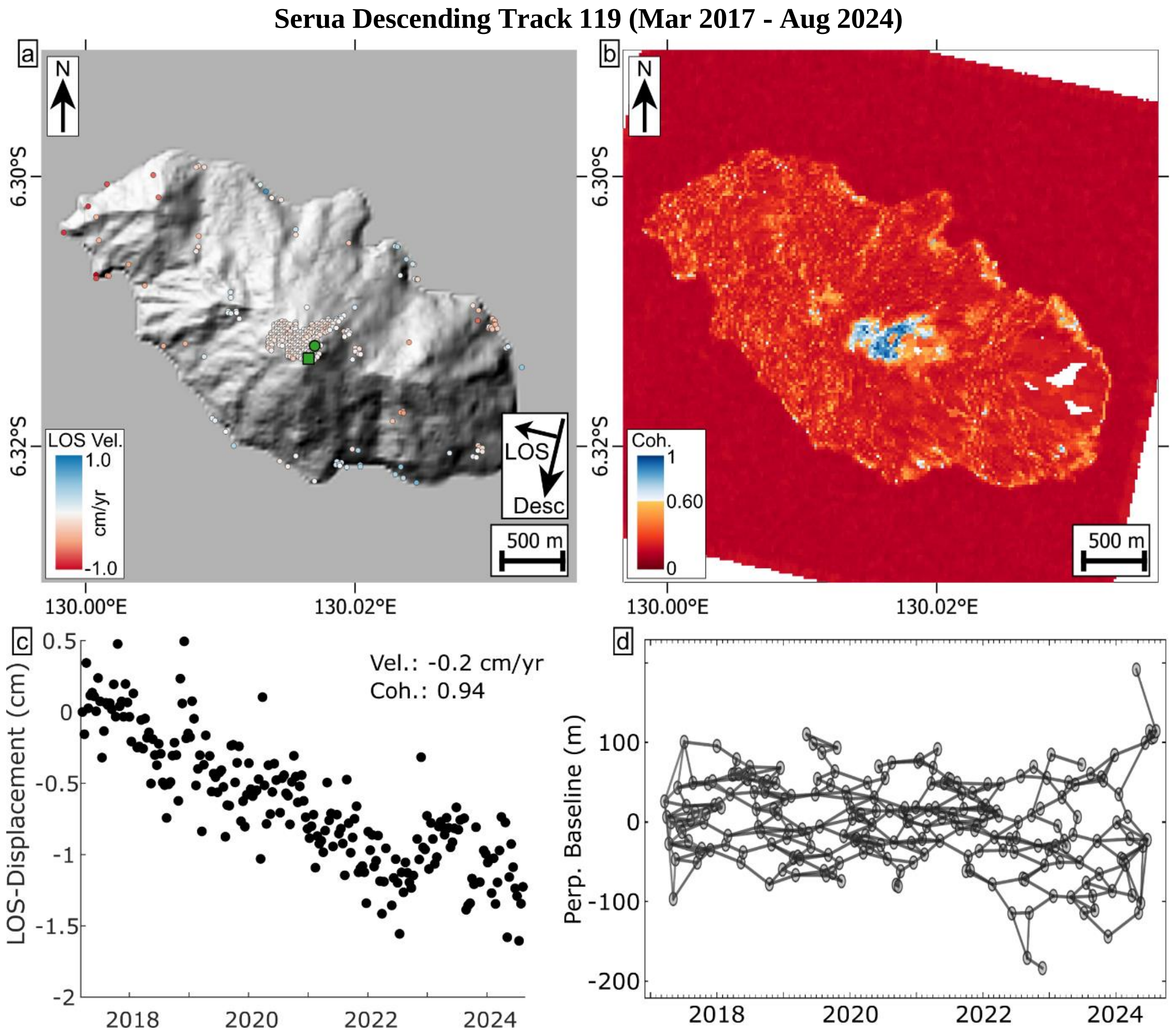

Figure S22: LOS-Timeseries InSAR data for Serua volcano, Indonesia, in descending orbit showing (a) the LOS-velocity map on a hillshade background. The green square marks the reference point and the green circle marks the selected timeseries point plotted in (c), (b) the temporal coherence map, (c) the selected point time series with the average velocity and the coherence for the selected point, and (d) the interferogram connections network plot.

Sirung Ascending Track 39 (Jan 2017 - Nov 2024)

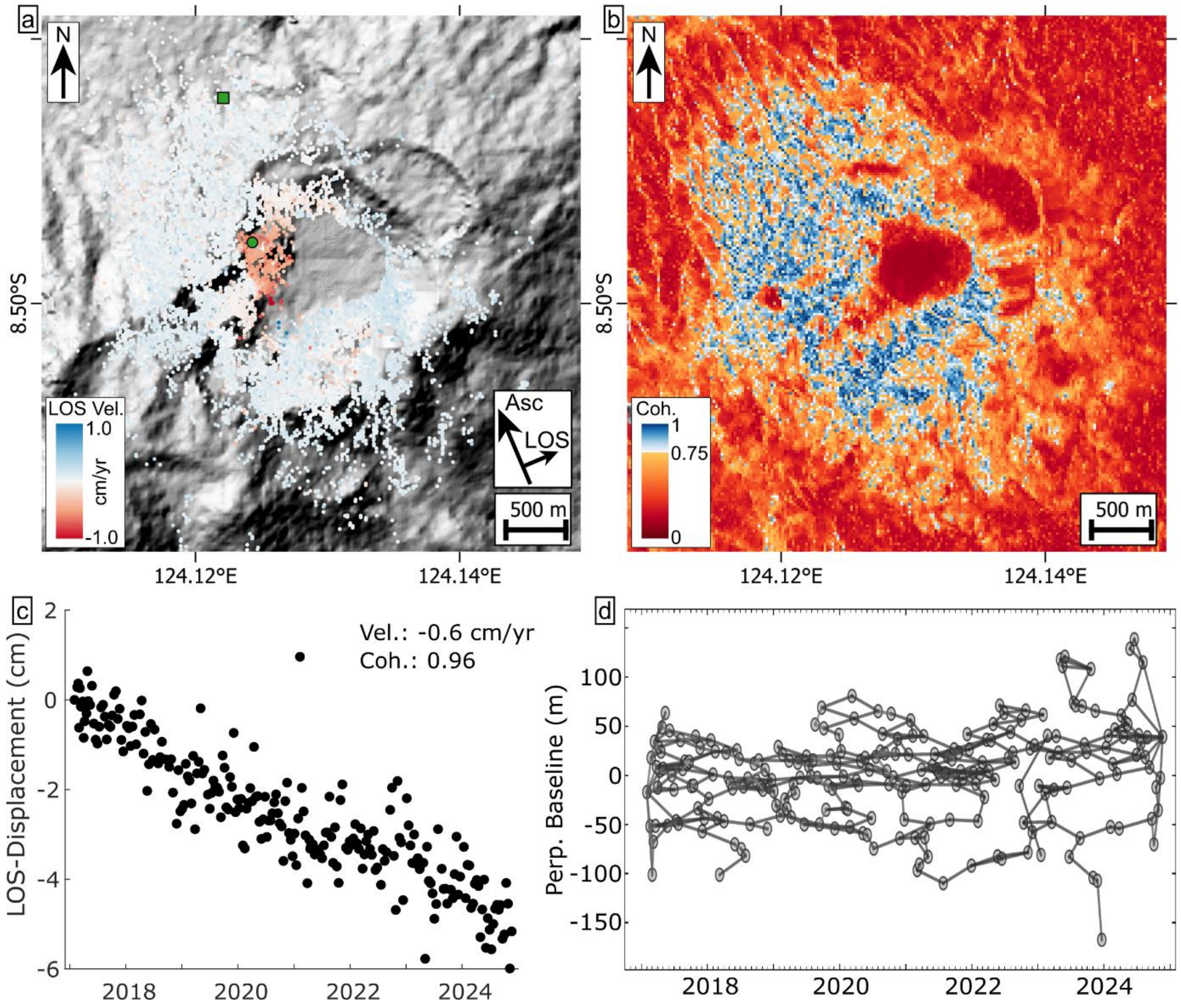

Figure S23: LOS-Timeseries InSAR data for Sirung volcano, Indonesia, in ascending orbit showing (a) the LOS-velocity map on a hillshade background. The green square marks the reference point and the green circle marks the selected timeseries point plotted in (c), (b) the temporal coherence map, (c) the selected point time series with the average velocity and the coherence for the selected point, and (d) the interferogram connections network plot.

Sirung Descending Track 134 (Jan 2017 - Nov 2024)

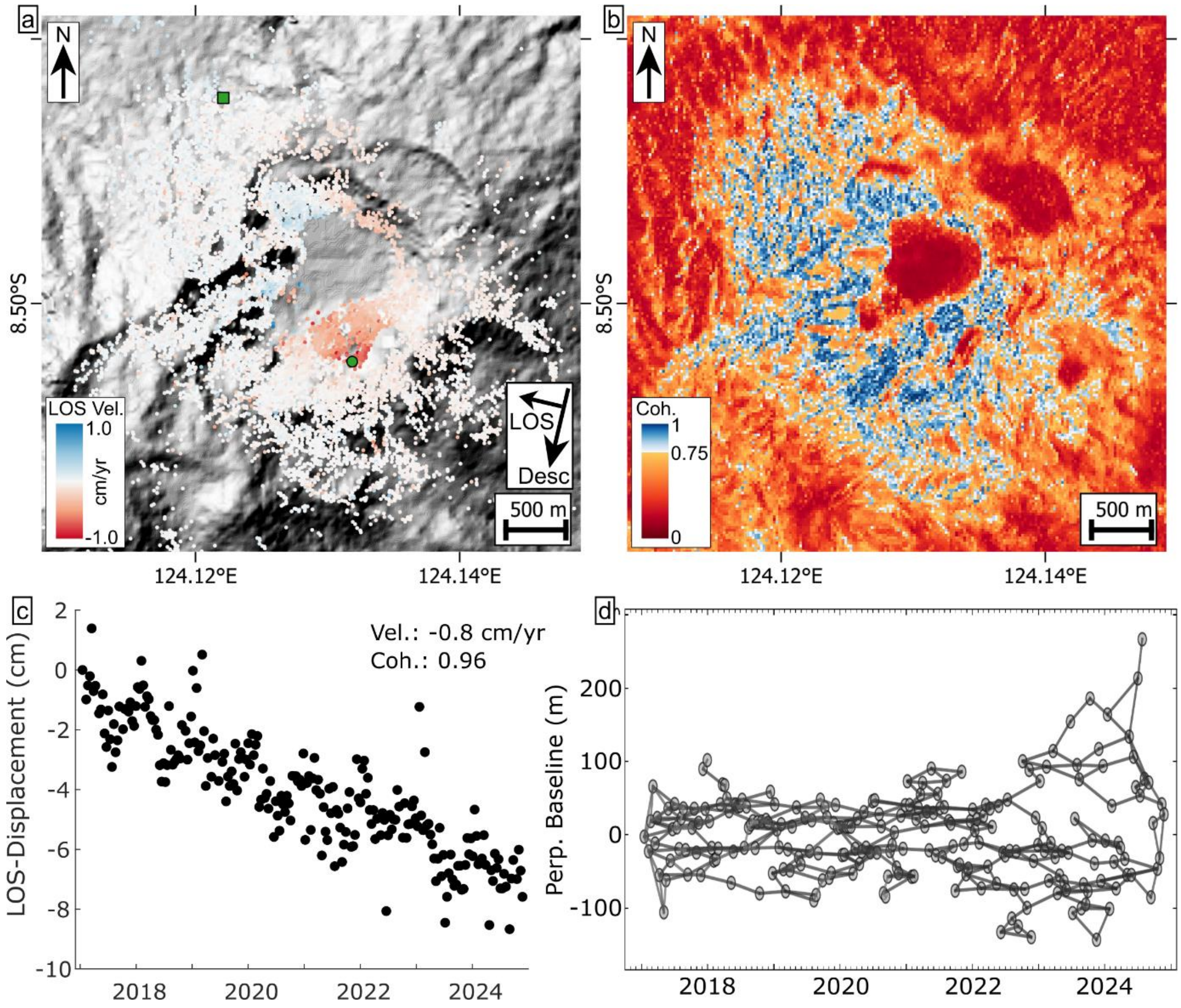

Figure S24: LOS-Timeseries InSAR data for Sirung volcano, Indonesia, in descending orbit showing (a) the LOS-velocity map on a hillshade background. The green square marks the reference point and the green circle marks the selected timeseries point plotted in (c), (b) the temporal coherence map, (c) the selected point time series with the average velocity and the coherence for the selected point, and (d) the interferogram connections network plot.

Ulawun Ascending Track 67 (Mar 2016 - Aug 2024)

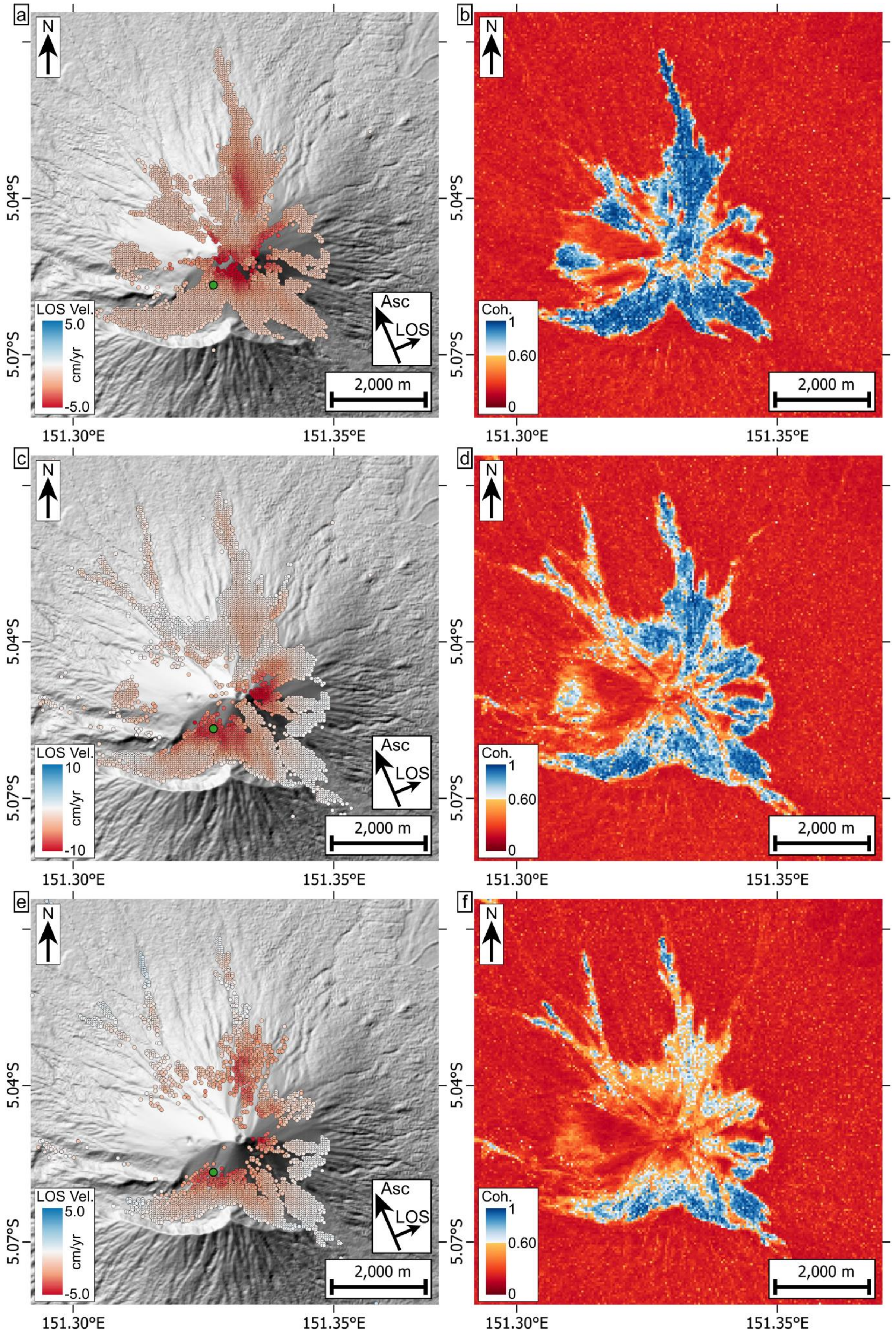

Figure S25: LOS-Timeseries InSAR data (ascending) for Ulawun (continued below).

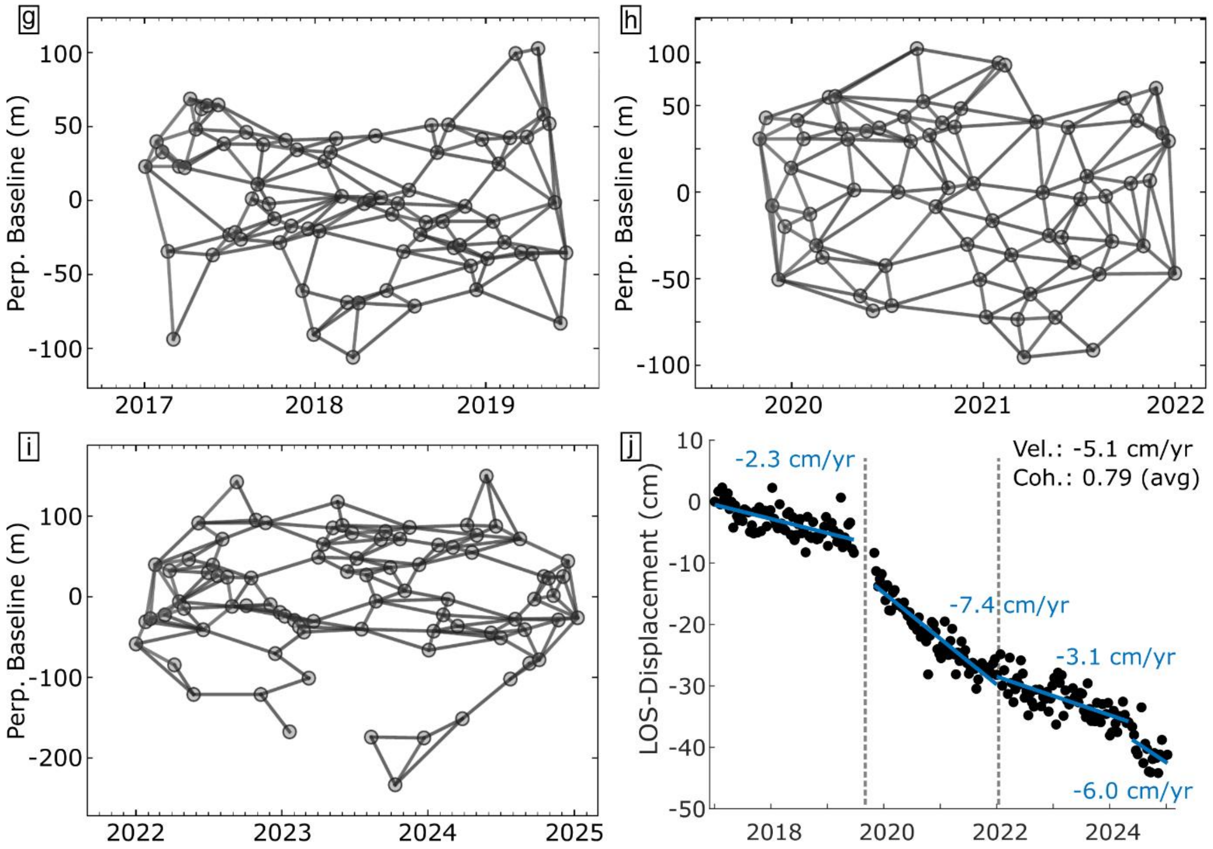

Figure S25 (continued): LOS-Timeseries InSAR data for Ulawun volcano, Papua New Guinea, in ascending orbit showing (a) the LOS-velocity map on a hillshade background. The green square marks the reference point and the green circle marks the selected timeseries point plotted in (c), (b) the temporal coherence map, (c) the selected point time series with the average velocity and the coherence for the selected point, and (d) the interferogram connections network plot.

Ulawun Descending Track 16 (Jan 2017 - Aug 2024)

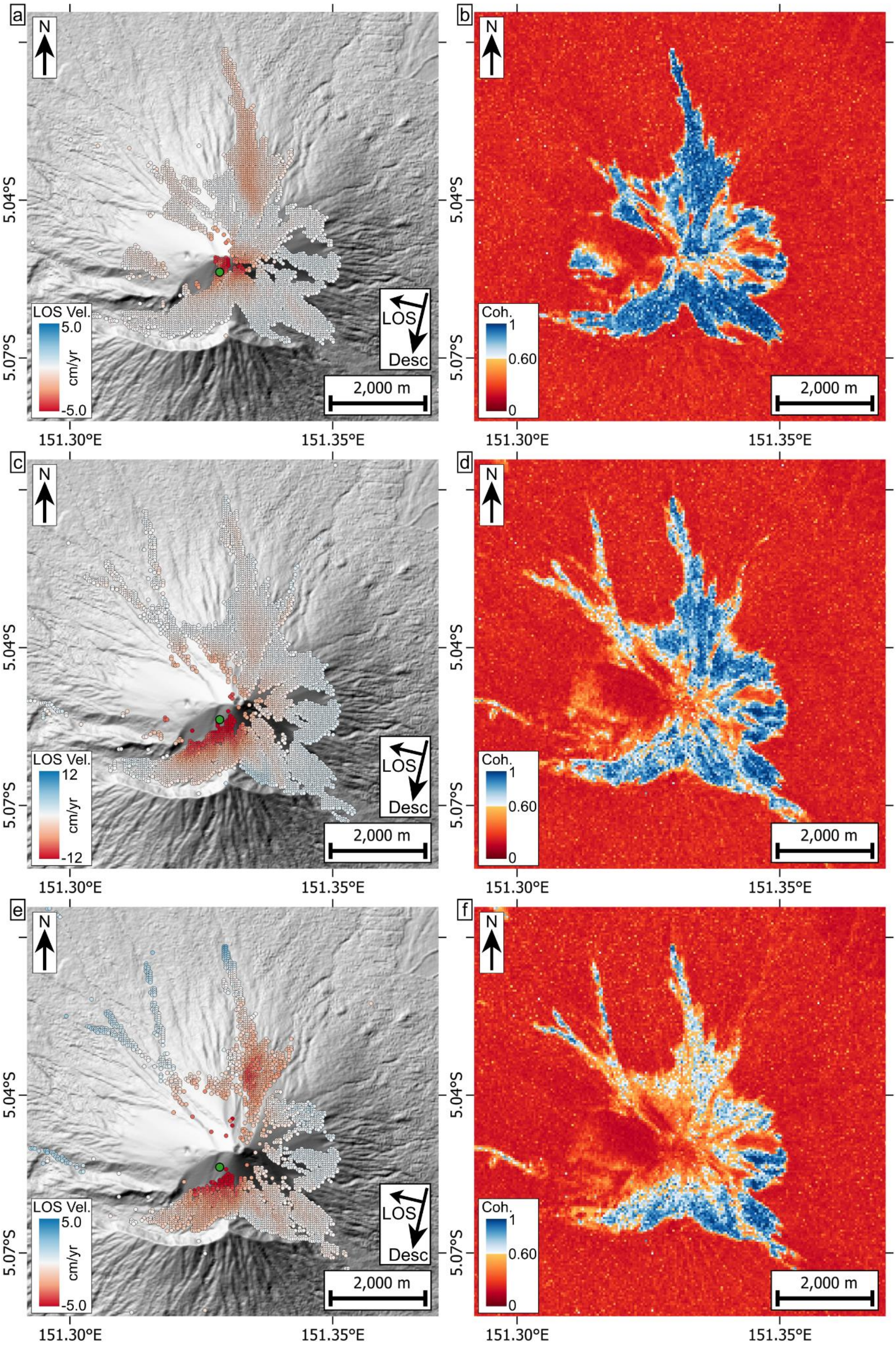

Figure S26: LOS-Timeseries InSAR data (descending) for Ulawun (continued below).

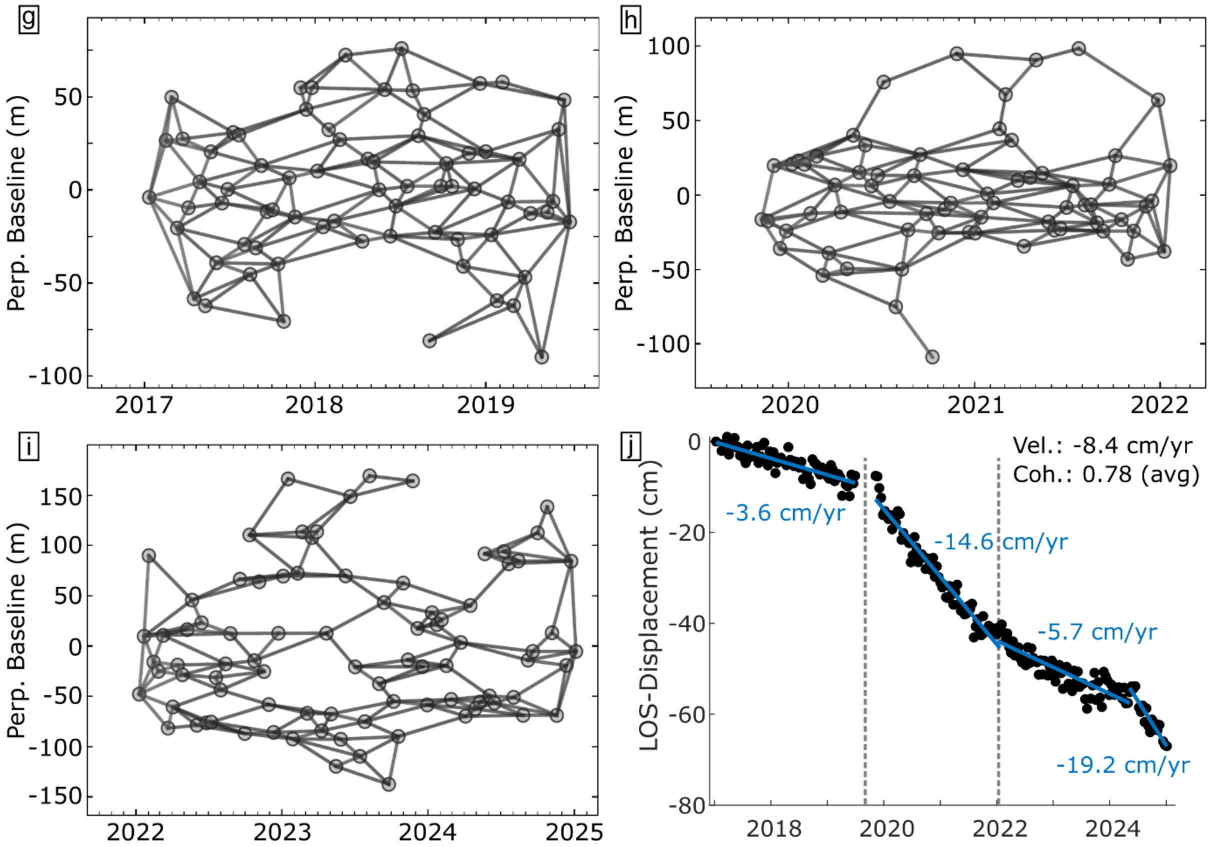

Figure S26 (continued): LOS-Timeseries InSAR data for Ulawun volcano, Papua New Guinea, in descending orbit showing (a) the LOS-velocity map on a hillshade background. The green square marks the reference point and the green circle marks the selected timeseries point plotted in (c), (b) the temporal coherence map, (c) the selected point time series with the average velocity and the coherence for the selected point, and (d) the interferogram connections network plot.

Wetar Descending Track 17 (Jan 2027 - Aug 2024)

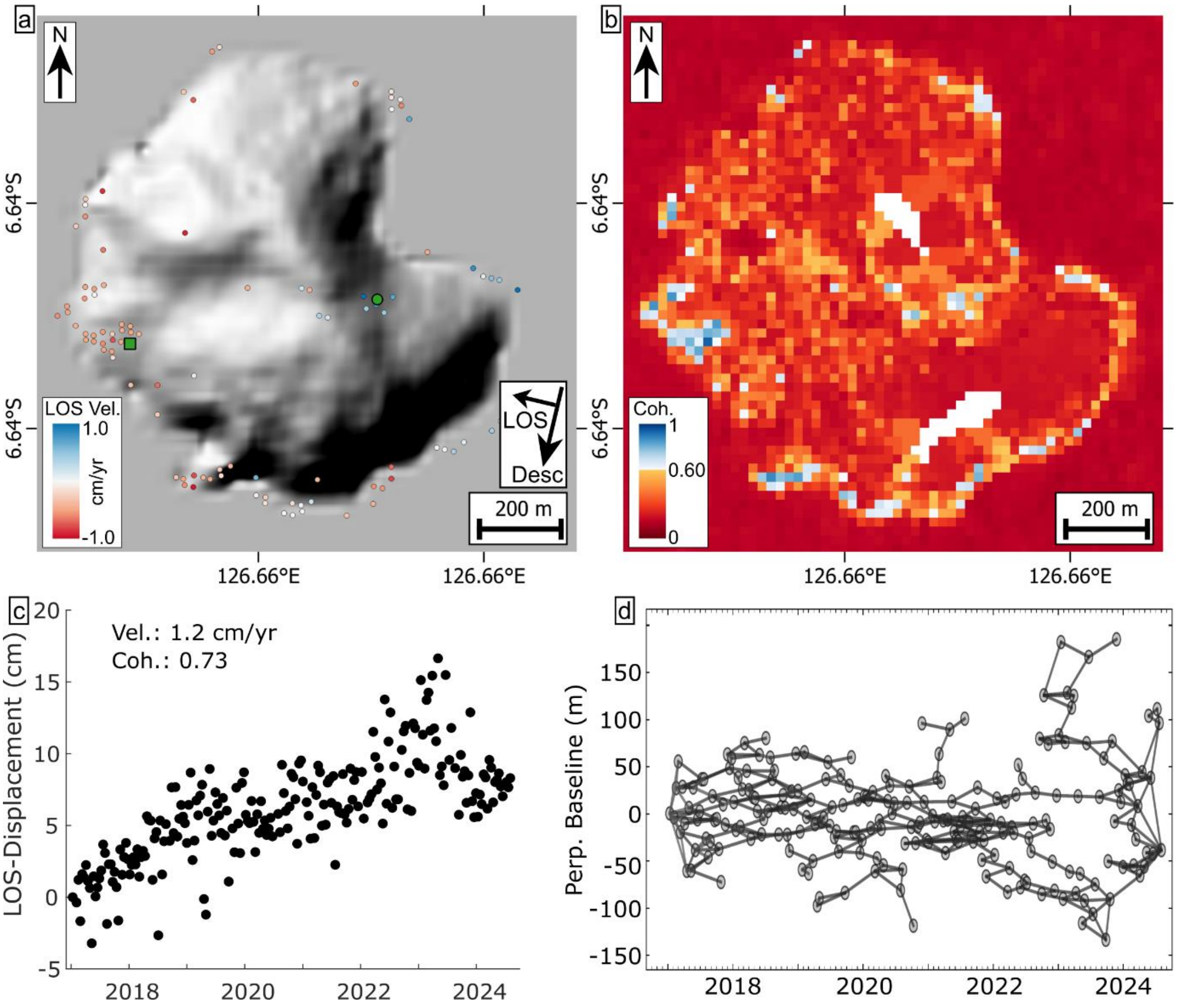

Figure S27: LOS-Timeseries InSAR data for Wetar volcano, Indonesia, in descending orbit showing (a) the LOS-velocity map on a hillshade background. The green square marks the reference point and the green circle marks the selected timeseries point plotted in (c), (b) the temporal coherence map, (c) the selected point time series with the average velocity and the coherence for the selected point, and (d) the interferogram connections network plot.

No known activity or historical eruptions are known from this small and remote volcanic island. Due to its small size (<1 km diameter) and near full vegetation, hardly any coherent points are present. Therefore, the data is highly susceptible to unwrapping errors and it is unlikely that any significant deformation could be detected. The data here suggests very minor subsidence on the western side and minor uplift on the eastern side of the island, however, this may be due to the arbitrary choice of reference point. Therefore, we deem the deformation data from Wetar to be unreliable and it should be interpreted with caution.
